# COVID-19 vaccine safety: Background incidence rates of anaphylaxis, myocarditis, pericarditis, Guillain-Barré Syndrome, and mortality in South Korea using a nationwide population-based cohort study

**DOI:** 10.1371/journal.pone.0297902

**Published:** 2024-02-21

**Authors:** Hye Su Jeong, Byung Chul Chun

**Affiliations:** 1 Drug Safety Monitoring Center, National Medical Center, Seoul, South Korea; 2 Department of Public Health, Korea University Graduate School, Seoul, South Korea; 3 Department of Preventive Medicine, Korea University College of Medicine, Seoul, South Korea; 4 Department of Epidemiology and Health Informatics Graduate School of Public Health, Korea University, Seoul, South Korea; Korea Disease Control and Prevention Agency, REPUBLIC OF KOREA

## Abstract

**Background:**

To properly assess an association between vaccines and specific adverse events requires a comparison between the observed and background rates; however, studies in South Korea are currently limited. Therefore, in this study, we estimated the background incidence of anaphylaxis, myocarditis, pericarditis, Guillain-Barré syndrome (GBS), and mortality in South Korea.

**Methods:**

A retrospective cohort study was conducted using the National Sample Cohort (NSC) data. Using NSC, the background incidence rate was estimated by dividing the number of episodes during 2009–2019 by the total population by year and then multiplying by 100,000. Using Statistics Korea data, the background mortality rate was estimated by dividing the number of deaths, during 2009–2019 by the standard population for that year and then multiplying by 100,000. Using background mortality rates, we predicted mortality rates for 2021 using autoregressive integrated moving average models. Further, the expected mortality rates were compared with observed mortality rates.

**Results:**

The age-adjusted incidence rate (AIR) of anaphylaxis increased from 4.28 to 22.90 cases per 100,000 population (p = 0.003); myocarditis showed no significant increase, changing from 0.56 to 1.26 cases per 100,000 population (p = 0.276); pericarditis increased from 0.94 to 1.88 cases per 100,000 population (p = 0.005); and GBS increased from 0.78 to 1.21 cases per 100,000 population (p = 0.013). The age-adjusted mortality rate decreased from 645.24 to 475.70 deaths per 100,000 population (p <0.001). The 2021 observed/expected mortality rates for overall (ratio: 1.08, 95% confidence interval [CI]: 1.07–1.08), men (ratio: 1.07, 95% CI: 1.07–1.08), and women (ratio: 1.08, 95% CI: 1.07–1.09), were all significantly higher. When stratified by age group, those aged ≥80 (ratio: 1.16, 95% CI: 1.15–1.17), 60–69 (ratio: 1.11, 95% CI: 1.10–1.13), and 20–29 years old (ratio: 1.07, 95% CI: 1.02–1.13) were also significantly higher.

**Conclusion:**

Through the estimation of background rates related to anaphylaxis, myocarditis, pericarditis, GBS, and mortality, we established a reference point for evaluating the potential excess occurrence of adverse events following COVID-19 vaccination. This reference point serves as substantive evidence supporting the safety profile of COVID-19 vaccines.

## 1. Introduction

Controlling the outbreak of SARS-CoV-2 heavily relies on the acceptance of COVID-19 vaccines [1]. According to a survey conducted in South Korea prior to the introduction of COVID-19 vaccines, 39.8% of respondents were hesitant to receive the vaccine, and among them, for 77.9% this was due to concerns about vaccine adverse events (AEs) and safety [2]. Establishing evidence of safety can increase public acceptance of COVID-19 vaccines [3]. This evidence can be established based on the background rate of rare but serious AEs confirmed through pre-market clinical trials [4], and post-marketing surveillance of AEs that may occur following vaccination [5].

In the USA, a systematic review was conducted to estimate the background rates of 22 adverse events of special interest (AESI), including anaphylaxis, myocarditis, pericarditis, Guillain-Barré syndrome (GBS), and death [5]. In Japan, a study was conducted to estimate the background rates of 43 events, including anaphylaxis, myocarditis, pericarditis, GBS, and death, prior to the introduction of COVID-19 vaccines [6]. In eight countries, including Australia, France, and Japan, using 13 databases, the background rates of 15 AESI, including GBS, myocarditis/pericarditis, and stroke, were estimated [7].

One study compared the observed and background rates of GBS after vaccination using the Vaccine Adverse Event Reporting System (VAERS) in the US to suggest an association with the Ad26.COV2.S (Janssen) vaccine [8]. Another study used the Vaccine Safety Datalink (VSD), a US healthcare database, to also compare the observed and background rates of GBS after vaccination to suggest an association of GBS with the Janssen vaccine [9]. Conversely, one study showed no association with mRNA COVID-19 vaccines by comparing the observed and background rates of thrombocytopenia after vaccination using data from the VAERS [10]. To properly assess an association between vaccines and specific AEs requires a comparison between the observed and background rates; however, studies in South Korea are currently limited. Therefore, the aim of this study was to estimate the background rates of hospitalized anaphylaxis, myocarditis, pericarditis, GBS, and mortality in South Korea during eleven prepandemic years from 2009 to 2019. Additionally, the study sought to compare the observed mortality rate in 2021 with the expected mortality rate.

## 2. Material and methods

### 2.1. Database

#### 2.1.1. Background incidence rate

This retrospective study made use of data obtained from the National Sample Cohort (NSC), established by the South Korean National Health Insurance Service (NHIS). In order to ensure the representativeness of the entire population, NHIS employed a systematic stratified random sampling approach with proportional allocation based on gender, age, type of insurance, income quintiles, and geographic region. Approximately 2.1% of the estimated total population, equivalent to around 1 million, was thoughtfully selected to comprise the NSC [11]. This study included a population from NSC between 2009 and 2019, and data were accessed on December 2022.

#### 2.1.2. Background mortality rate

We collected data on the number of deaths from the publicly available Statistics Korea [12]. This study included a population between 2009 and 2019, and data were accessed on December 2022.

### 2.2. Operational definition

The events for which background rates could be estimated using the NSC were selected from among the AESIs in the adverse reactions after COVID-19 vaccination in South Korea, distributed by the Korea Disease Control and Prevention Agency [13]. The operational definition was reviewed by clinicians and epidemiologists.

#### 2.2.1. Anaphylaxis

Using the NSC, we identified patients who were hospitalized for anaphylaxis based on the 10th revision of the International Classification Disease (ICD-10) codes as the primary diagnosis. Cases with clearly identifiable external causes were excluded, as in previous studies [14, 15]. If a patient had multiple visits for anaphylaxis, we considered them as separate episodes if the interval between the last date of the first visit and the first date of the second visit was at least 28 days [16]. Detailed codes are listed in S1 Table.

#### 2.2.2. Myocarditis/pericarditis

Using the NSC, we identified patients who were hospitalized with myocarditis [17–20] and pericarditis [20, 21] based on ICD-10 codes as the primary diagnosis. If a patient had multiple visits for myocarditis or pericarditis, we considered them as separate episodes if the interval between the last date of the first visit and the first date of the second visit was at least 365 days [22, 23]. Detailed codes are listed in S1 Table.

#### 2.2.3. GBS

Using the NSC, we defined patients who were hospitalized with GBS based on ICD-10 codes [24] as the primary diagnosis and the relieved co-payment policy code for GBS. The relieved co-payment policy is a South Korean program that reduces the payment rate for health insurance and requires a diagnosis by a neurology specialist [25]. If a patient had multiple visits with GBS, we considered them as separate episodes if the interval between the last date of the first visit and the first date of the second visit was at least 60 days [26]. Detailed codes are listed in S1 Table.

### 2.3. Statistical analysis

The background incidence rate was estimated by dividing the number of episodes during 2009–2019 by the total population in the NSC by year and then multiplying by 100,000. The background mortality rate was estimated by dividing the number of deaths, excluding those of unknown age, during 2009–2019 by the standard population of that year and then multiplying by 100,000. Age-adjusted incidence and mortality rates were estimated for the 2015 standard population using the direct standardization method. The 95% confidence interval (CI) was estimated using a Poisson distribution.

Using the background mortality rates from to 2009–2019, autoregressive integrated moving average models were used to predict the expected mortality rates for 2021, using the "forecast" package and "auto.arima" function in R software [27]. Further, the expected mortality rates were compared with observed mortality rates.

The results were considered significant at a significance level of 95% with a p-value <0.05. The statistical software used was R software version 3.3 (R Foundation for Statistical Computing, Vienna, Austria) and SAS v9.4 (SAS Institute, Cary, NC, USA).

### 2.4. Ethical statement

The study was approved by institutional review board of Korea University (KUIRB-2022-0059). As unidentified and anonymized information was applied for analysis, the need for informed consent was waived.

## 3. Results

### 3.1. Background incidence, mortality rate

During the study period, there were a total of 1,041 cases of anaphylaxis, with men (548 cases, 52.6%) comprising a higher proportion than women (493 cases, 47.4%). When stratified by age groups, the most frequent occurrences were in the 50–59 age group (258 cases, 24.8%), followed by the 60–69 age group (185 cases, 17.8%), and the 40–49 age group (174 cases, 16.7%) (S2 Table). The crude incidence rate (CIR) of anaphylaxis, as observed in S3 Table, demonstrates a rising trend from 4.15 cases in 2009 to 22.06 cases per 100,000 population in 2019 (p-value = 0.003). When stratified by gender, the incidence rate was mostly higher in men than in women and when categorized by age groups, individuals aged 50 and above consistently had higher incidence rates. The age-adjusted incidence rate (AIR) of anaphylaxis increased from 4.28 cases per 100,000 population in 2009 to 22.90 cases per 100,000 population in 2019 (p = 0.003). Except for in 2012 (men: 3.72, women: 3.95), men had higher rates than women in all years (Table 1).

**Table 1:** Trends of anaphylaxis age-adjusted incidence rate in 2009–2019.

Year	Total (95% CI)	Men (95% CI)	Women (95% CI)
**2009**	4.28 (4.10–4.46)	4.53 (4.35–4.71)	4.03 (3.85–4.20)
**2010**	4.31 (4.13–4.49)	4.96 (4.77–5.16)	3.66 (3.50–3.83)
**2011**	4.04 (3.86–4.21)	4.54 (4.36–4.73)	3.53 (3.37–3.69)
**2012**	3.84 (3.67–4.01)	3.72 (3.56–3.89)	3.95 (3.78–4.12)
**2013**	6.09 (5.87–6.30)	7.05 (6.82–7.28)	5.13 (4.93–5.32)
**2014**	6.03 (5.81–6.24)	6.60 (6.38–6.83)	5.45 (5.25–5.65)
**2015**	4.60 (4.42–4.79)	5.58 (5.37–5.78)	3.63 (3.47–3.80)
**2016**	14.68 (14.34–15.01)	19.07 (18.69–19.45)	10.29 (10.01–10.57)
**2017**	20.36 (19.97–20.76)	20.71 (20.32–21.11)	20.01 (19.63–20.40)
**2018**	21.70 (21.30–22.11)	24.61 (24.18–25.04)	18.80 (18.43–19.18)
**2019**	22.90 (22.48–23.32)	24.49 (24.06–24.92)	21.31 (20.91–21.71)
**p for trend**	0.003	0.003	0.008

CI: confidence interval

The incidence rate of anaphylaxis is expressed in episodes per 100,000 population.

Age adjusted to the 2015 South Korean standard population by direct standardization method.

During the study period, there were 81 cases of myocarditis, with men (53 cases, 65.4%) comprising a higher proportion than women (28 cases, 34.6%). When stratified by age groups, the most frequent occurrences were in the 0–19 age group (32 cases, 39.5%), followed by the 20–29 age group (13 cases, 16.0%), and the 30–39 and 40–49 age groups (each with 10 cases, 12.3%) (S4 Table). The CIR of myocarditis, as observed in S5 Table, did not demonstrate a rising trend from 0.61 cases in 2009 to 0.65 cases per 100,000 population in 2019 (p-value = 0.533). When stratified by gender, the incidence rate was mostly similar or higher in men than in women and when categorized by age groups the incidence rate was higher in relatively younger age groups (below aged 40). The AIR of myocarditis did not show a significant increase, changing from 0.56 cases per 100,000 population in 2009 to 1.26 cases per 100,000 population in 2019 (p = 0.276). Except for in 2009 (men: 0.55, women: 0.57), 2018 (men: 0.59, Women:1.07), and 2019 (men: 1.00, women:1.51), men had higher rates than women (Table 2).

**Table 2:** Trends of myocarditis age-adjusted incidence rate in 2009–2019.

Year	Total (95% CI)	Men (95% CI)	Women (95% CI)
**2009**	0.56 (0.50–0.63)	0.55 (0.49–0.62)	0.57 (0.51–0.64)
**2010**	0.48 (0.42–0.54)	0.59 (0.52–0.66)	0.36 (0.31–0.41)
**2011**	0.93 (0.85–1.02)	1.48 (1.37–1.58)	0.39 (0.34–0.44)
**2012**	0.71 (0.64–0.78)	0.90 (0.82–0.99)	0.51 (0.45–0.58)
**2013**	1.48 (1.38–1.59)	2.29 (2.16–2.42)	0.68 (0.61–0.76)
**2014**	0.85 (0.77–0.93)	1.51 (1.40–1.62)	0.19 (0.16–0.23)
**2015**	0.55 (0.49–0.62)	0.92 (0.83–1.00)	0.19 (0.16–0.23)
**2016**	1.38 (1.27–1.48)	1.97 (1.85–2.09)	0.78 (0.71–0.86)
**2017**	1.30 (1.20–1.40)	1.56 (1.45–1.67)	1.03 (0.95–1.12)
**2018**	0.83 (0.75–0.91)	0.59 (0.53–0.66)	1.07 (0.98–1.16)
**2019**	1.26 (1.16–1.35)	1.00 (0.91–1.09)	1.51 (1.41–1.62)
**p for trend**	0.276	0.276	0.029

CI: confidence interval

The incidence rate of myocarditis is expressed in episodes per 100,000 population.

Age adjusted to the 2015 South Korean standard population by direct standardization method.

During the study period, there were 133 cases of pericarditis, with men (78 cases, 58.6%) comprising a higher proportion than women (55 cases, 41.4%). When stratified by age groups, it was observed that 49.6% of all patients were concentrated in the 60 years and older age group, with 60–69 years (26 cases, 19.5%), 70–79 years (21 cases, 15.8%), and 80 years and above (19 cases, 14.3%) being the most frequent age groups (S6 Table). The CIR of pericarditis, as observed in S7 Table, demonstrates a rising trend from 0.91 cases in 2009 to 1.83 cases per 100,000 population in 2019 (p-value = 0.003). When stratified by gender, the incidence rate was mostly higher in men than in women and when categorized by age groups the incidence rate was higher in relatively older age groups (aged 60 above). The AIR of pericarditis showed an increase from 0.94 cases per 100,000 population in 2009 to 1.88 cases per 100,000 population in 2019 (p = 0.005). Except for in 2017 (men: 1.45, women: 1.93), men had higher rates than women in all years (Table 3).

**Table 3:** Trends of pericarditis age-adjusted incidence rate in 2009–2019.

Year	Total (95% CI)	Men (95% CI)	Women (95% CI)
**2009**	0.94 (0.86–1.02)	1.02 (0.94–1.11)	0.86 (0.78–0.94)
**2010**	0.56 (0.49–0.62)	0.90 (0.81–0.98)	0.22 (0.18–0.26)
**2011**	1.24 (1.14–1.33)	1.87 (1.75–1.99)	0.61 (0.54–0.67)
**2012**	0.84 (0.76–0.92)	0.90 (0.81–0.98)	0.79 (0.71–0.87)
**2013**	1.02 (0.93–1.11)	1.07 (0.98–1.16)	0.97 (0.89–1.06)
**2014**	1.15 (1.06–1.25)	1.55 (1.44–1.65)	0.76 (0.69–0.84)
**2015**	1.00 (0.92–1.09)	1.09 (1.00–1.18)	0.92 (0.84–1.00)
**2016**	1.49 (1.38–1.60)	2.30 (2.16–2.43)	0.68 (0.61–0.76)
**2017**	1.69 (1.58–1.81)	1.45 (1.35–1.56)	1.93 (1.81–2.05)
**2018**	1.88 (1.77–2.01)	2.39 (2.26–2.52)	1.38 (1.28–1.49)
**2019**	1.88 (1.76–2.00)	2.79 (2.64–2.93)	0.97 (0.88–1.05)
**p for trend**	0.005	0.008	0.087

CI: confidence interval

The incidence rate of pericarditis is expressed in episodes per 100,000 population.

Age adjusted to the 2015 South Korean standard population by direct standardization.

During the study period, there were 162 cases of GBS, with men (88 cases, 54.3%) comprising a higher proportion than women (74 cases, 45.7%). When stratified by age groups, the most frequent occurrences were in the 50–59 age group (32 cases, 19.8%), followed by the 40–49 age group (31 cases, 19.1%), and the 60–69 age group (23 cases, 14.2%) (S8 Table). The CIR of GBS, as observed in S9 Table, demonstrates a rising trend from 0.81 cases in 2009 to 1.51 cases per 100,000 population in 2019 (p-value = 0.002). When stratified by gender, the incidence rate was mostly higher in men than in women and when categorized by age groups the incidence rate was relatively higher in aged 40 and above. The AIR of GBS increased from 0.78 cases per 100,000 population in 2009 to 1.21 cases per 100,000 population in 2019 (p = 0.013). Except for in 2011 (men: 0.86, women: 1.07), 2013 (men: 1.06, women: 1.56), 2015 (men: 0.74, women: 2.06), and 2016 (men: 2.05, women: 2.22), men had higher rates than women in all years (Table 4).

**Table 4:** Trends of Guillain-Barré syndrome age-adjusted incidence rate in 2009–2019.

Year	Total (95% CI)	Men (95% CI)	Women (95% CI)
**2009**	0.78 (0.70–0.86)	0.95 (0.87–1.04)	0.61 (0.54–0.68)
**2010**	1.13 (1.04–1.22)	1.22 (1.12–1.31)	1.04 (0.95–1.13)
**2011**	0.96 (0.88–1.05)	0.86 (0.78–0.94)	1.07 (0.98–1.16)
**2012**	1.35 (1.25–1.45)	1.64 (1.53–1.75)	1.05 (0.96–1.14)
**2013**	1.31 (1.21–1.41)	1.06 (0.94–1.15)	1.56 (1.46–1.67)
**2014**	1.69 (1.58–1.80)	1.91 (1.79–2.03)	1.47 (1.37–1.58)
**2015**	1.40 (1.30–1.50)	0.74 (0.66–0.81)	2.06 (1.94–2.18)
**2016**	2.14 (2.01–2.26)	2.05 (1.93–2.17)	2.22 (2.10–2.36)
**2017**	1.89 (1.78–2.02)	2.67 (2.53–2.82)	1.12 (1.02–1.21)
**2018**	2.19 (2.06–2.32)	2.96 (2.81–3.11)	1.42 (1.31–1.52)
**2019**	1.21 (1.12–1.31)	1.47 (1.37–1.58)	0.95 (0.86–1.03)
**p for trend**	0.013	0.062	0.213

CI: confidence interval

The incidence rate of GBS is expressed in episodes per 100,000 population.

Age adjusted to the 2015 South Korean standard population by direct standardization method.

During the study period, there were 2,996,598 deaths, with men (1,643,195 deaths, 54.8%) comprising a higher proportion than women (1,353,403 deaths, 45.2%). When stratified by age groups, the most frequent cases were observed in the 80 years and above age group (1,192,280 deaths, 39.8%), followed by the 70–79 age group (780,735 deaths, 26.1%), and the 60–69 age group (417,212 deaths, 13.9%) (S10 Table). The crude mortality rate, as observed in S11 Table, demonstrates a rising trend from 497.20 deaths in 2009 to 574.71 deaths per 100,000 population in 2019 (p-value<0.001). When stratified by gender, the incidence rate was higher in men than in women. When categorized by age groups, the mortality rate was highest in those aged 80 and above, followed by the 70–79 age group, and then the 60–69 age group. The age-adjusted mortality rate showed a decrease from 645.24 deaths per 100,000 population in 2009 to 475.70 deaths per 100,000 population in 2019 (p <0.001). Men showed higher rates than women in all years (Table 5).

**Table 5:** Trends of age-adjusted mortality rate in 2009–2019.

Year	Total (95% CI)	Men (95% CI)	Women (95% CI)
**2009**	645.24 (643.03–647.44)	717.46 (715.14–719.79)	573.11 (571.03–575.19)
**2010**	636.78 (634.59–638.98)	708.37 (706.06–710.68)	565.30 (563.24–567.36)
**2011**	613.88 (611.73–616.04)	682.72 (680.46–684.99)	545.14 (543.11–547.17)
**2012**	607.26 (605.12–609.40)	669.08 (666.83–671.32)	545.53 (543.50–547.56)
**2013**	575.09 (573.01–577.17)	632.71 (630.53–634.90)	517.55 (515.57–519.53)
**2014**	551.43 (549.39–553.47)	606.82 (604.68–608.96)	496.12 (494.19–498.05)
**2015**	541.4 (539.38–543.42)	590.89 (588.77–593.00)	491.99 (490.06–493.92)
**2016**	525.33 (523.34–527.32)	571.18 (569.11–573.26)	479.54 (477.64–481.44)
**2017**	508.14 (506.19–510.10)	549.38 (547.34–551.41)	466.96 (465.09–468.84)
**2018**	505.87 (503.92–507.83)	544.88 (542.85–546.91)	466.92 (465.04–468.80)
**2019**	475.70 (473.81–477.60)	515.48 (513.51–517.45)	435.98 (434.17–437.80)
**p for trend**	<0.001	<0.001	<0.001

CI: confidence interval

The mortality rate is expressed per 100,000 population.

Age adjusted to the 2015 South Korean standard population by direct standardization method.

### 3.2. Observed/expected mortality rate

The total number of deaths in 2021 was 317,655, with an overall mortality rate of 618.81 deaths per 100,000 population. The rate was higher in men (672.02 per 100,000) than women (565.92 per 100,000).

As presented in Table 6, when comparing the observed mortality rates with the expected mortality rates for 2021, the overall (ratio: 1.08, 95% CI: 1.07–1.08), men (ratio: 1.07, 95% CI: 1.07–1.08) and women (ratio: 1.08, 95% CI: 1.07–1.09), were all found to be statistically significantly higher. By age group, the rates were statistically significantly higher for those aged 80 years and over (ratio: 1.16, 95% CI: 1.15–1.17), 60–69 years (ratio: 1.11, 95% CI: 1.10–1.13), and 20–29 years (ratio: 1.07, 95% CI: 1.02–1.13).

**Table 6:** Observed/expected rate ratio of death in 2021.

	Observed rate	Expected rate	Observed rate/expected rate	
			ratio	95% CI
**Total**	618.81	574.84	1.08	(1.07–1.08)
**Gender**				
Men	672.02	626.61	1.07	(1.07–1.08)
Women	565.92	523.42	1.08	(1.07–1.09)
**Age group**				
0–19	19.59	21.87	0.90	(0.84–0.96)
20–29	41.43	38.54	1.07	(1.02–1.13)
30–39	67.24	67.25	1.00	(0.96–1.04)
40–49	137.65	148.54	0.93	(0.90–0.95)
50–59	297.52	319.36	0.93	(0.92–0.95)
60–69	646.21	580.04	1.11	(1.10–1.13)
70–79	1,873.59	1,916.88	0.98	(0.97–0.99)
80+	7,847.28	6,773.85	1.16	(1.15–1.17)

Observed rate is based on based on Statistics Korea death data per 100,000 population, expected rate was predicted mortality rate of 2021 based on 2009–2019 background rate using autoregressive integrated moving average model.

CI: confidence interval

## 4. Discussion

This study estimated the background incidence rates of anaphylaxis, myocarditis, pericarditis, and GBS from 2009 to 2019 using the NSC data. Further, background mortality rates during the same period were estimated using data from Statistics Korea. Based on these data, the expected mortality rates for 2021 were estimated and compared with the observed mortality rates.

The AIR of anaphylaxis analyzed in this study showed an increasing trend, from 4.28 to 22.90 cases per 100,000 population. This aligns with the upward trends observed in different regions. For instance, there was an increase from 16.02 to 32.19 cases per 100,000 person-years (py) over a 7-year period (2008–2014) based on health insurance claims data for hospital visitors in South Korea, which includes outpatient and inpatient [14]. Similarly, there was a rise from 4.79 to 8.20 cases per 100,000 py over a 13-year period (2001–2013) based on health insurance claims data for hospital visitors in Taiwan, which includes outpatient, inpatient, emergency department, and intensive care unit [28]. Additionally, an analysis of 20 years (1992–2012) of hospitalization data in the UK revealed an increase from 1.0 to 7.0 cases per 100,000 population [29]. The difference in incidence rate compared with previous studies could be due to variations in the operational definition or differences in the timing of the study. In this study, men showed a higher incidence rate than women in most cases, which is consistent with findings from studies in South Korea [14], and Taiwan [28]. However, a study conducted in the UK reported a higher incidence rate in women than in men [29]. The role of female sex hormones in allergic diseases, such as asthma and rhinitis, is known to affect T cells and B cells, but the pathogenesis of anaphylaxis according to sex has not been clearly elucidated [30].

In South Korea in 2022, COVID-19 vaccines in use include Pfizer, Moderna, ChAdOx1 (AstraZeneca), Janssen, and NVX-CoV2373 (Novavax) vaccines. Following COVID-19 vaccination, the mechanism of anaphylaxis is not clear; however, it has been suggested that polyethylene glycol (PEG), which is used to stabilize lipid nanoparticles in mRNA COVID-19 vaccines, can cause allergic reactions [31, 32]. Additionally, polysorbate 80, an adjuvant in ChAdOx1 (AstraZeneca), Janssen, and NVX-CoV2373 (Novavax) vaccines, can cause hypersensitivity reactions and is structurally related to PEG, which may result in cross-reactivity and anaphylaxis [33].

The AIR of myocarditis analyzed in this study was 1.26 cases per 100,000 population in 2019. In Spain, the incidence rates in 2017 among hospitalized patients were 2.0 cases per 100,000 population [34], and hospitalized patients in Sweden, among patients registered over 15 years (2000–2014) was 8.6 cases per 100,000 population in those aged ≥16 years [19]. In this study, men had a higher incidence rate than women in most cases, which is consistent with the results of a study conducted in Sweden [19]. The pathogenesis of myocarditis according to sex is unclear; however, it is known that sex hormones have a direct effect on cardiac function, endothelial cell function, and vascular tone, and thus may influence the mechanisms of myocardial cell damage [35].

The AIR of pericarditis analyzed in this study showed an increasing trend, from 0.94 to 1.88 cases per 100,000 population. This was lower than the incidence rate of pericarditis analyzed in 29 hospitals in Finland among hospitalized patients during 2000–2009, which was 3.32 cases per 100,000 py [36]. In this study, men had a higher incidence rate than women in most cases, which is consistent with the results of a Finnish study [36]. The pathogenesis of pericarditis according to sex is not clear, but it is known that women are more likely to be underdiagnosed with pericarditis and that there are differences depending on sex hormones [37]. Progesterone worsens cardiac inflammation [38], and it is known that the risk of pericarditis increases owing to decreased levels of estrogen after menopause [36].

According to an analysis of data collected from the VAERS between December 2020 and August 2021, statistically significant rates of myocarditis and pericarditis were observed with mRNA COVID-19 vaccines compared with viral vector COVID-19 vaccines [39]. The mechanism of myocarditis and pericarditis after COVID-19 vaccination is not clear; however, it has been suggested that immune reactions to mRNA vaccines and uncontrolled cytokine expression may be involved [40].

The AIR of GBS analyzed in this study showed an increasing trend, from 0.78 to 1.21 cases per 100,000 population. This pattern aligned with the rise in hospitalized patients in South Korea, as indicated by health insurance claims data spanning 7 years (2010–2016), which showed an increase from 1.28 to 1.82 cases per 100,000 population [24]. A similar trend was noted hospitalized patients in Taiwan, from health insurance claims data over 5 years (1997–2011), with an increase from 1.52 to 2.1 cases per 100,000 py [41]. However, hospitalized patients in Denmark, the incidence rate over 30 years (1987–2016) using Danish national registry data revealed no increasing trends, remaining relatively stable from 1.70 to 1.76 cases per 100,000 population [42]. In this study, men showed a higher incidence rate than women in most cases, which is consistent with the results of studies in South Korea [24], Taiwan [41], and Denmark [42]. Additionally, an analysis of hospital visitors, which includes emergency department and inpatient, with VSD over a 10-year period (2000–2009) indicated that the incidence rate was higher in men than in women, with rates of 3.70 cases per 100,000 py for men and 2.64 cases for women [43]. The pathogenesis of GBS according to sex is unclear; however, GBS is known to have a protective effect of estrogen, unlike other autoimmune diseases [44].

The mechanism of GBS following COVID-19 vaccination is not clear; however, it has been suggested that the immune response to antibody production after vaccination may induce an autoimmune response, leading to the generation of antibodies against myelin, which can result in GBS [45].

In Norway, 23 deaths were reported following BNT162b2 (Pfizer) vaccination, and it was reported that some patients experienced common AEs, such as fever and nausea after mRNA COVID-19 vaccination, which could lead to death in some cases [46]. In Qatar, deaths following Pfizer vaccination between December 2020 and March 2021 were more frequently reported in elderly patients with comorbidities, such as diabetes and hypertension [47]. According to a study in Japan, 1,368 deaths have been reported after COVID-19 vaccination. However, no causal relationship has been established between the reported deaths and vaccines [48].

In comparing the observed and expected mortality rates in South Korea in 2021, the overall (ratio: 1.08, 95% CI: 1.07–1.08) was found to be significantly higher. It is difficult to interpret the observed increase in mortality as solely attributable to COVID-19 vaccination. Despite COVID-19 vaccination, breakthrough infections have occurred owing to the emergence of various viral variants [49], and it cannot be ruled out that some deaths may have been caused by SARS-CoV-2 infection.

The limitations of this study were as follows. First, there were limitations to the claims data. For the NSC, which was used to estimate the background incidence rate as collected for billing purposes, the possibility of misclassification of the diagnosis codes cannot be excluded [50]. Second, there are limitations to the data period. As with the comparison of observed and expected mortality rates, others should also be compared; however, because the NSC data were only available up to 2019, it was difficult to compare them with the 2021 observed rates. Third, there were some limitations to the methodology. Although background rates are required to evaluate vaccine safety, comparing background rates with observed rates only suggests the possibility of an association between the vaccine and AEs and does not reveal a causal relationship [3].

The strengths of this study were as follows: First, we used NSC and Statistics Korea data, which provide representation of the entire South Korean population to estimate the background rates of potential AEs following COVID-19 vaccination. Second, to the best of our knowledge, this is the first study to estimate the background incidence rate using the NSC and to compare the observed/expected mortality rate in 2021 in South Korea.

## 5. Conclusion

We estimated the background incidence of anaphylaxis, myocarditis, pericarditis, GBS, and mortality in South Korea. When comparing the observed and expected mortality rates for 2021, the overall mortality rates for those aged 80 years and older, those aged 60–69 years, and those aged 20–29 years were found to be significantly higher. Through the estimation of background rates related to anaphylaxis, myocarditis, pericarditis, GBS, and mortality, we established a reference point for evaluating the potential excess occurrence of adverse events following COVID-19 vaccination. This reference point serves as substantive evidence supporting the safety profile of COVID-19 vaccines.

## Supporting information

S1 Checklist STROBE statement—checklist of items that should be included in reports of observational studies. (DOCX)

S1 Table Operational definition of events by ICD-10 code. (DOCX)

S2 Table Demographic characteristic of anaphylaxis cases. (DOCX)

S3 Table Crude incidence rate of anaphylaxis in 2009–2019. (DOCX)

S4 Table Demographic characteristic of myocarditis cases. (DOCX)

S5 Table Crude incidence rate of myocarditis in 2009–2019. (DOCX)

S6 Table Demographic characteristic of pericarditis cases. (DOCX)

S7 Table Crude incidence rate of pericarditis in 2009–2019. (DOCX)

S8 Table Demographic characteristic of Guillain–Barrè syndrome cases. (DOCX)

S9 Table Crude incidence rate of Guillain–Barrè syndrome (GBS) in 2009–2019. (DOCX)

S10 Table Demographic characteristic of death. (DOCX)

S11 Table Crude mortality rate in 2009–2019. (DOCX)

## References

[1] Sallam M. COVID-19 Vaccine Hesitancy Worldwide: A Concise Systematic Review of Vaccine Acceptance Rates. Vaccines. 2021;9(2):160. doi: 10.3390/vaccines9020160 33669441 PMC7920465

[2] Hwang SE, Kim W-H, Heo J. Socio-demographic, psychological, and experiential predictors of COVID-19 vaccine hesitancy in South Korea, October-December 2020. Hum Vaccin Immunother. 2022;18(1):1–8. doi: 10.1080/21645515.2021.1983389 34614382 PMC8920123

[3] Black SB, Law B, Chen RT, Dekker CL, Sturkenboom M, Huang WT, et al. The critical role of background rates of possible adverse events in the assessment of COVID-19 vaccine safety. Vaccine. 2021;39(19):2712–2718. doi: 10.1016/j.vaccine.2021.03.016 33846042 PMC7936550

[4] Wormsbecker AE, Johnson C, Bourns L, Harris T, Crowcroft NS, Deeks SL. Demonstration of background rates of three conditions of interest for vaccine safety surveillance. PLoS One. 2019;14(1):e0210833. doi: 10.1371/journal.pone.0210833 30645649 PMC6333343

[5] Gubernot D, Jazwa A, Niu M, Baumblatt J, Gee J, Moro P, et al. U.S. Population-Based background incidence rates of medical conditions for use in safety assessment of COVID-19 vaccines. Vaccine. 2021;39(28):3666–3677. doi: 10.1016/j.vaccine.2021.05.016 34088506 PMC8118666

[6] Sobue T, Fukuda H, Matsumoto T, Lee B, Ito S, Iwata S. The background occurrence of selected clinical conditions prior to the start of an extensive national vaccination program in Japan. PLoS One. 2021;16(8):e0256379. doi: 10.1371/journal.pone.0256379 34437567 PMC8389412

[7] Li X, Ostropolets A, Makadia R, Shaoibi A, Rao G, Sena AG, et al. Characterizing the incidence of adverse events of special interest for COVID-19 vaccines across eight countries: a multinational network cohort study. medRxiv. 2021; doi: 10.1101/2021.03.25.21254315 35727911 PMC8193077

[8] Woo EJ, Mba-Jonas A, Dimova RB, Alimchandani M, Zinderman CE, Nair N. Association of Receipt of the Ad26.COV2.S COVID-19 Vaccine With Presumptive Guillain-Barre Syndrome, February-July 2021. JAMA. 2021;326(16):1606–1613. doi: 10.1001/jama.2021.16496 34617967 PMC8498927

[9] Hanson KE, Goddard K, Lewis N, Fireman B, Myers TR, Bakshi N, et al. Incidence of Guillain-Barré Syndrome After COVID-19 Vaccination in the Vaccine Safety Datalink. JAMA Netw Open. 2022;5(4):e228879. doi: 10.1001/jamanetworkopen.2022.8879 35471572 PMC9044108

[10] Welsh KJ, Baumblatt J, Chege W, Goud R, Nair N. Thrombocytopenia including immune thrombocytopenia after receipt of mRNA COVID-19 vaccines reported to the Vaccine Adverse Event Reporting System (VAERS). Vaccine. 2021;39(25):3329–3332. doi: 10.1016/j.vaccine.2021.04.054 34006408 PMC8086806

[11] NHIS. Guide to sample cohort DB. [cited 14 March 2023]. In: NHIS web sites [Internet]. Available from: https://nhiss.nhis.or.kr/bd/ab/bdaba002cv.do

[12] Statistics Korea. Korean statisitcal information service. [cited 15 March 2023]. In: Statistics Korea web sites [Internet]. Available from: https://kosis.kr/eng/

[13] KCDA. Trends in occurrence of adverse reactions in Korea. [cited 14 March 2023]. In: KCDA websites [Internet]. Available from: https://ncv.kdca.go.kr/pot/bbs/BD_selectBbsList.do?q_bbsSn=1018

[14] Yang MS, Kim JY, Kim BK, Park HW, Cho SH, Min KU, et al. True rise in anaphylaxis incidence: Epidemiologic study based on a national health insurance database. Medicine (Baltimore). 2017;96(5):e5750. doi: 10.1097/MD.0000000000005750 28151851 PMC5293414

[15] Ye Y, Kim M, Kang H, Kim T, Sohn S, Koh Y, et al. Predictors of the Severity and Serious Outcomes of Anaphylaxis in Korean Adults: A Multicenter Retrospective Case Study. Allergy Asthma Immunol Res. 2015;7(1):22. doi: 10.4168/aair.2015.7.1.22 25553259 PMC4274465

[16] Clothier HJ, Lee KJ, Sundararajan V, Buttery JP, Crawford NW. Human papillomavirus vaccine in boys: background rates of potential adverse events. Med J Aust. 2013;198(10):554–558. doi: 10.5694/mja12.11751 23725271

[17] Kim J, Cho M. Acute Myocarditis in Children: a 10-year Nationwide Study (2007–2016) based on the Health Insurance Review and Assessment Service Database in Korea. Korean Circ J. 2020;50(11):1013. doi: 10.4070/kcj.2020.0108 32812406 PMC7596206

[18] Mevorach D, Anis E, Cedar N, Bromberg M, Haas EJ, Nadir E, et al. Myocarditis after BNT162b2 mRNA Vaccine against Covid-19 in Israel. N Engl J Med. 2021;385(23):2140–2149. doi: 10.1056/NEJMoa2109730 34614328 PMC8531987

[19] Fu M, Kontogeorgos S, Thunstrom E, Zverkova Sandstrom T, Kroon C, Bollano E, et al. Trends in myocarditis incidence, complications and mortality in Sweden from 2000 to 2014. Sci Rep. 2022;12(1):1810. doi: 10.1038/s41598-022-05951-z 35110692 PMC8810766

[20] Park H, Yun KW, Kim K-R, Song SH, Ahn B, Kim DR, et al. Epidemiology and Clinical Features of Myocarditis/Pericarditis before the Introduction of mRNA COVID-19 Vaccine in Korean Children: a Multicenter Study. J Korean Med Sci. 2021;36(32):e232. doi: 10.3346/jkms.2021.36.e232 34402230 PMC8369310

[21] Husby A, Hansen JV, Fosbøl E, Thiesson EM, Madsen M, Thomsen RW, et al. SARS-CoV-2 vaccination and myocarditis or myopericarditis: population based cohort study. BMJ. 2021;375(375):e068665. doi: 10.1136/bmj-2021-068665 34916207 PMC8683843

[22] Chua GT, Kwan MYW, Chui CSL, Smith RD, Cheung EC-L, Ma T, et al. Epidemiology of Acute Myocarditis/Pericarditis in Hong Kong Adolescents Following Comirnaty Vaccination. CID. 2022;75(4): 673–681. doi: 10.1093/cid/ciab989 34849657 PMC8767823

[23] Oster ME, Shay DK, Su JR, Gee J, Creech CB, Broder KR, et al. Myocarditis Cases Reported After mRNA-Based COVID-19 Vaccination in the US From December 2020 to August 2021. JAMA. 2022;327(4):331. doi: 10.1001/jama.2021.24110 35076665 PMC8790664

[24] Kim A, Lee H, Lee Y, Kang H. Epidemiological Features and Economic Burden of Guillain-Barré Syndrome in South Korea: A Nationwide Population-Based Study. J Clin Neurol. 2021;17(2):257. doi: 10.3988/jcn.2021.17.2.257 33835747 PMC8053545

[25] NHIS. Relieved co-payment policy. [cited 15 March 2023]. In: NHIS web sites [Internet]. Availale from: https://www.nhis.or.kr/static/html/wbma/c/wbmac0215.html

[26] Kwong JC, Vasa PP, Campitelli MA, Hawken S, Wilson K, Rosella LC, et al. Risk of Guillain-Barré syndrome after seasonal influenza vaccination and influenza health-care encounters: a self-controlled study. Lancet Infect Dis. 2013;13(9):769–776. doi: 10.1016/S1473-3099(13)70104-X 23810252

[27] Hyndman R, Athanasopoulos G, Bergmeir C, Caceres G, Chhay L, O’Hara-Wild M, et al. Forecasting Functions for Time Series and Linear Models forecast. [cited 15 March 2023]. In: forecast web sites [Internet]. Available from:https://pkg.robjhyndman.com/forecast/

[28] Yao T-C, Wu AC, Huang Y-W, Wang J-Y, Tsai H-J. Increasing trends of anaphylaxis-related events: an analysis of anaphylaxis using nationwide data in Taiwan, 2001–2013. World Allergy Organ J. 2018;11(1):23. doi: 10.1186/s40413-018-0202-7 30349617 PMC6178262

[29] Turner PJ, Gowland MH, Sharma V, Ierodiakonou D, Harper N, Garcez T, et al. Increase in anaphylaxis-related hospitalizations but no increase in fatalities: an analysis of United Kingdom national anaphylaxis data, 1992–2012. J Allergy Clin Immunol. 2015;135:956–963. doi: 10.1016/j.jaci.2014.10.021 25468198 PMC4382330

[30] Salvati L, Vitiello G, Parronchi P. Gender differences in anaphylaxis. Curr Opin Allergy Clin Immunol. 2019;19(5):417–424. doi: 10.1097/ACI.0000000000000568 31465313

[31] Garvey LH, Nasser S. Anaphylaxis to the first COVID-19 vaccine: is polyethylene glycol (PEG) the culprit? Br J Anaesth. 2021;126(3):e106–e108. doi: 10.1016/j.bja.2020.12.020 33386124 PMC7834677

[32] Sellaturay PA-O, Nasser SA-O, Islam S, Gurugama P, Ewan PA-O. Polyethylene glycol (PEG) is a cause of anaphylaxis to the Pfizer/BioNTech mRNA COVID-19 vaccine. Clin Exp Allergy. 2021;(51(6)):861–863. doi: 10.1111/cea.13874 33825239 PMC8251011

[33] Barbaud A, Garvey LH, Arcolaci A, Brockow K, Mori F, Mayorga C, et al. Allergies and COVID‐19 vaccines: an ENDA/EAACI position paper. Allergy. 2022;77(8):2292–2312. doi: 10.1111/all.15241 35112371

[34] Willame C, Dodd C, Gini R, Durán C, Thomsen R, Wang L, et al. Background rates of Adverse Events of Special Interest for monitoring COVID-19 vaccines. Zenodo. 2021; doi: 10.5281/zenodo.5255870

[35] Fairweather D, Cooper LT, Blauwet LA. Sex and Gender Differences in Myocarditis and Dilated Cardiomyopathy. Curr Probl Cardiol. 2013;38(1):7–46. doi: 10.1016/j.cpcardiol.2012.07.003 23158412 PMC4136454

[36] Kytö V, Sipilä J, Rautava P. Clinical profile and influences on outcomes in patients hospitalized for acute pericarditis. Circulation. 2014;130(18):1601–1606. doi: 10.1161/CIRCULATIONAHA.114.010376 25205801

[37] Laufer-Perl M, Havakuk O, Shacham Y, Steinvil A, Letourneau-Shesaf S, Chorin E, et al. Sex-based differences in prevalence and clinical presentation among pericarditis and myopericarditis patients. Am J Emerg Med. 2017;35(2):201–205. doi: 10.1016/j.ajem.2016.10.039 27836311

[38] Lyden DC, Huber SA. Aggravation of coxsackievirus, group B, type 3-induced myocarditis and increase in cellular immunity to myocyte antigens in pregnant Balb/c mice and animals treated with progesterone. Cell Immunol. 1984;87(2):462–472. doi: 10.1016/0008-8749(84)90015-7 6088088

[39] Li M, Yuan J, Lv G, Brown J, Jiang X, Lu ZK. Myocarditis and Pericarditis following COVID-19 Vaccination: Inequalities in Age and Vaccine Types. J Pers Med. 2021;11(11):1106. doi: 10.3390/jpm11111106 34834458 PMC8624452

[40] Bozkurt B, Kamat I, Hotez PJ. Myocarditis With COVID-19 mRNA Vaccines. Circulation. 2021;144(6):471–484. doi: 10.1161/CIRCULATIONAHA.121.056135 34281357 PMC8340726

[41] Huang W-C, Lu C-L, Chen SC-C. A 15-year nationwide epidemiological analysis of Guillain-Barré syndrome in Taiwan. Neuroepidemiology. 2015;44(4):249–254. doi: 10.1159/000430917 26088600

[42] Levison LS, Thomsen RW, Christensen DH, Mellemkjær T, Sindrup SH, Andersen H. Guillain-Barré syndrome in Denmark: validation of diagnostic codes and a population-based nationwidestudy of the incidence in a 30-year period. Clin Epidemiol. 2019:275–283. doi: 10.2147/clep.s199839 31114387 PMC6497480

[43] Shui IM, Rett MD, Weintraub E, Marcy M, Amato AA, Sheikh SI, et al. Guillain-Barré Syndrome Incidence in a Large United States Cohort (2000–2009). Neuroepidemiology. 2012;39(2):109–115. doi: 10.1159/000339248 22846726

[44] Flachenecker P. Epidemiology of neuroimmunological diseases. J Neurol. 2006;253(S5):v2–v8. doi: 10.1007/s00415-006-5001-3 16998750

[45] Meylan S, Livio F, Foerster M, Genoud PJ, Marguet F, Wuerzner G. Stage III Hypertension in Patients After mRNA-Based SARS-CoV-2 Vaccination. Hypertension. 2021;77(6):e56–e57. doi: 10.1161/HYPERTENSIONAHA.121.17316 33764160 PMC8115421

[46] Torjesen I. Covid-19: Norway investigates 23 deaths in frail elderly patients after vaccination. BMJ. 2021:n149. doi: 10.1136/bmj.n149 33451975

[47] Zaqout A, Daghfal J, Alaqad I, Hussein SAN, Aldushain A, Almaslamani MA, et al. The initial impact of a national BNT162b2 mRNA COVID-19 vaccine rollout. Int J Infect Dis. 2021;108:116–118. doi: 10.1016/j.ijid.2021.05.021 33992763 PMC8117665

[48] Yamaguchi T, Iwagami M, Ishiguro C, Fujii D, Yamamoto N, Narisawa M, et al. Safety monitoring of COVID-19 vaccines in Japan. Lancet Reg Health West Pac. 2022;23:100442. doi: 10.1016/j.lanwpc.2022.100442 35359913 PMC8960038

[49] Gupta RK, Topol EJ. COVID-19 vaccine breakthrough infections. Science. 2021;374(6575):1561–1562. doi: 10.1126/science.abl8487 34941414

[50] Lee J, Lee JS, Park S-H, Shin SA, Kim K. Cohort Profile: The National Health Insurance Service–National Sample Cohort (NHIS-NSC), South Korea. Int J Epidemiol. 2016:dyv319. doi: 10.1093/ije/dyv319 26822938

