# Supplementary material for: COVID-19 vaccine safety: Background incidence rates of anaphylaxis, myocarditis, pericarditis, Guillain-Barré Syndrome, and mortality in South Korea using a nationwide population-based cohort study
Source: PLoS One. 2024 Feb 21;19(2):e0297902. doi: 10.1371/journal.pone.0297902 (PMC10881009; doi:10.1371/journal.pone.0297902)
Supplement: S1 Checklist — (DOCX) [file pone.0297902.s001.docx]

STROBE Statement—checklist of items that should be included in reports of observational studies

|  | Item No. | Recommendation | Page  No. | Relevant text from manuscript |
| --- | --- | --- | --- | --- |
| **Title and abstract** | 1 | (*a*) Indicate the study’s design with a commonly used term in the title or the abstract | 1, 2 | (Title) COVID-19 vaccine safety: Background incidence rates of anaphylaxis, myocarditis, pericarditis, Guillain-Barré Syndrome, and mortality in South Korea using a nationwide population-based cohort study  (Abstract-methods) A retrospective cohort study was conducted using the National Sample Cohort (NSC) data. |
|  |  | (*b*) Provide in the abstract an informative and balanced summary of what was done and what was found | 2 | We estimated the background incidence rate of anaphylaxis, myocarditis, pericarditis, Guillain-Barré syndrome (GBS), and mortality in South Korea. Using background mortality rates, we predicted mortality rates for 2021 using autoregressive integrated moving average models. Further, the expected mortality rates were compared with observed mortality rates. |
| Introduction | | | |  |
| Background/rationale | 2 | Explain the scientific background and rationale for the investigation being reported | 3 | To properly assess an association between vaccines and specific adverse events requires a comparison between the observed and background rates.  There were some studies estimating background rate of adverse events of special interest of COVID-19 vaccine. And to suggest association, some studies compared the observed and background rates of GBS after COVID-19 vaccination. |
| Objectives | 3 | State specific objectives, including any prespecified hypotheses | 3 | In this study, we estimated the background incidence of anaphylaxis, myocarditis, pericarditis, GBS, and mortality in South Korea. In order to eliminate the impact of the COVID-19 vaccine, which began its administration in 2021 in South Korea, the incidence rate for 2021 was predicted using background rate before vaccination. Furthermore, we compare the expected mortality rate with the observed mortality rate for 2021. |
| Methods | | | |  |
| Study design | 4 | Present key elements of study design early in the paper | 4 | This retrospective study was conducted from the National Sample Cohort (NSC). |
| Setting | 5 | Describe the setting, locations, and relevant dates, including periods of recruitment, exposure, follow-up, and data collection | 4 | The NSC from the National Health Insurance Service represents approximately 2.1% of the entire population (approximately 1 million) as of 2006. |
| Participants | 6 | (*a*) *Cohort study*—Give the eligibility criteria, and the sources and methods of selection of participants. Describe methods of follow-up  *Case-control study*—Give the eligibility criteria, and the sources and methods of case ascertainment and control selection. Give the rationale for the choice of cases and controls  *Cross-sectional study*—Give the eligibility criteria, and the sources and methods of selection of participants | 4 | The NSC from the National Health Insurance Service represents approximately 2.1% of the entire population (approximately 1 million) as of 2006. The cohort includes health insurance claims data from 2002 to 2019 and was stratified according to gender, age, type of insurance, income quintiles, and region. |
|  |  | (*b*) *Cohort study*—For matched studies, give matching criteria and number of exposed and unexposed  *Case-control study*—For matched studies, give matching criteria and the number of controls per case | n/a |  |
| Variables | 7 | Clearly define all outcomes, exposures, predictors, potential confounders, and effect modifiers. Give diagnostic criteria, if applicable | 4-5 | Outcome:  Background incidence rate (Anaphylaxis, Myocarditis/Pericarditis, GBS): Patients who were hospitalized based on ICD-10 codes as primary diagnosis. If a patient had multiple visits, we considered them as separate episodes if the interval between the last date of the first visit and the first date of the second visit was at least 28 (anaphylaxis), 365 (myocarditis/pericarditis), 60 (GBS) days. , Background mortality rate  Potential confounders: age |
| Data sources/ measurement | 8* | For each variable of interest, give sources of data and details of methods of assessment (measurement). Describe comparability of assessment methods if there is more than one group | 5 | Background incidence rate: No. of Episodes / total population in the NSC by year * 100,000  Background mortality rate: No. of deaths / standard population of that year*100,000 |
| Bias | 9 | Describe any efforts to address potential sources of bias | 4 | (Background incidence rate) The NSC from the National Health Insurance Service represents approximately 2.1% of the entire population (approximately 1 million) as of 2006. The cohort includes health insurance claims data from 2002 to 2019 and was stratified according to gender, age, type of insurance, income quintiles, and region.  (Background mortality rate) We collected data on the number of deaths from the publicly available Statistics Korea  The NSC data, along with Statistics Korea data, are considered representative of the South Korean population. |
| Study size | 10 | Explain how the study size was arrived at | n/a |  |

Continued on next page

| Quantitative variables | 11 | Explain how quantitative variables were handled in the analyses. If applicable, describe which groupings were chosen and why | n/a |  |
| --- | --- | --- | --- | --- |
| Statistical methods | 12 | (*a*) Describe all statistical methods, including those used to control for confounding | 5 | Background incidence rate: No. of Episodes / total population in the NSC by year * 100,000  Background mortality rate: No. of deaths / standard population of that year*100,000  Age-adjusted incidence and mortality rates were estimated for the 2015 standard population using the direct standardization method. The 95 % confidence interval (CI) was estimated using a Poisson distribution.  Using the background mortality rates from to 2009–2019, autoregressive integrated moving average models were used to predict the expected mortality rates for 2021, using the "forecast" package and "auto.arima" function in R software.  The results were considered significant at a significance level of 95% with a p-value <0.05. |
|  |  | (*b*) Describe any methods used to examine subgroups and interactions | n/a |  |
|  |  | (*c*) Explain how missing data were addressed | 5 | The background mortality rate was estimated by dividing the number of deaths, excluding those of unknown age. |
|  |  | (*d*) *Cohort study*—If applicable, explain how loss to follow-up was addressed  *Case-control study*—If applicable, explain how matching of cases and controls was addressed  *Cross-sectional study*—If applicable, describe analytical methods taking account of sampling strategy | n/a |  |
|  |  | (*e*) Describe any sensitivity analyses | n/a |  |
| Results | | | | |
| Participants | 13* | (a) Report numbers of individuals at each stage of study—eg numbers potentially eligible, examined for eligibility, confirmed eligible, included in the study, completing follow-up, and analysed | n/a |  |
|  |  | (b) Give reasons for non-participation at each stage | n/a |  |
|  |  | (c) Consider use of a flow diagram | n/a |  |
| Descriptive data | 14* | (a) Give characteristics of study participants (eg demographic, clinical, social) and information on exposures and potential confounders | 6-21 | [Anaphylaxis]  Total cases of 2009: 41  Men of 2009: 21  Women of 2009: 20  [Myocarditis]  Total cases of 2009: 6  Men of 2009: 3  Women of 2009: 3  [Pericarditis]  Total cases of 2009: 9  Men of 2009: 5  Women of 2009: 4  [GBS]  Total cases of 2009: 8  Men of 2009: 5  Women of 2009: 3  [Death]  Total cases of 2009: 246,894  Men of 2009: 137,701  Women of 2009: 109,193 |
|  |  | (b) Indicate number of participants with missing data for each variable of interest | n/a |  |
|  |  | (c) *Cohort study*—Summarise follow-up time (eg, average and total amount) | n/a |  |
| Outcome data | 15* | *Cohort study*—Report numbers of outcome events or summary measures over time | 6-21 | [Anaphylaxis]  The age-adjusted incidence rate (AIR) of anaphylaxis increased from 4.28 cases per 100,000 population in 2009 to 22.90 cases per 100,000 population in 2019 (p = 0.003).  [Myocarditis]  The AIR of myocarditis did not show a significant increase, changing from 0.56 cases per 100,000 population in 2009 to 1.26 cases per 100,000 population in 2019 (p = 0.276).  [Pericarditis]  The AIR of pericarditis showed an increase from 0.94 cases per 100,000 population in 2009 to 1.88 cases per 100,000 population in 2019 (p = 0.005).  [GBS]  The AIR of GBS increased from 0.78 cases per 100,000 population in 2009 to 1.21 cases per 100,000 population in 2019 (p = 0.013).  [Death]  The age-adjusted mortality rate showed a decrease from 645.24 deaths per 100,000 population in 2009 to 475.70 deaths per 100,000 population in 2019 (p <0.001). |
|  |  | *Case-control study—*Report numbers in each exposure category, or summary measures of exposure | n/a |  |
|  |  | *Cross-sectional study—*Report numbers of outcome events or summary measures | n/a |  |
| Main results | 16 | (*a*) Give unadjusted estimates and, if applicable, confounder-adjusted estimates and their precision (eg, 95% confidence interval). Make clear which confounders were adjusted for and why they were included | 6-21 | [Anaphylaxis]  Crude incidence rate of 2009: 4.15 (2.94-5.47)  Age-adjusted incidence rate of 2009: 4.28 (4.10-4.46)  [Myocarditis]  Crude incidence rate of 2009: 0.61 (0.20-1.11)  Age-adjusted incidence rate of 2009: 0.56 (0.50-0.63)  [Pericarditis]  Crude incidence rate of 2009: 0.91 (0.40-1.52)  Age-adjusted incidence rate of 2009: 0.94 (0.86-1.02)  [GBS]  Crude incidence rate of 2009: 0.81 (0.30-1.42)  Age-adjusted incidence rate of 2009: 0.78 (0.70-0.86)  [Death]  Crude mortality rate of 2009: 497.20 (495.24-499.16)  Age-adjusted mortality rate of 2009: 645.24 (643.03-647.44) |
|  |  | (*b*) Report category boundaries when continuous variables were categorized | 22 | Age group: 0-19, 20-29, 30-39, 40-49, 50-59, 60-69, 70-79, 80+ |
|  |  | (*c*) If relevant, consider translating estimates of relative risk into absolute risk for a meaningful time period | n/a |  |

Continued on next page

| Other analyses | 17 | Report other analyses done—eg analyses of subgroups and interactions, and sensitivity analyses | n/a |  |
| --- | --- | --- | --- | --- |
| Discussion | | | | |
| Key results | 18 | Summarise key results with reference to study objectives | 23 | This study estimated the background incidence rates of anaphylaxis, myocarditis, pericarditis, and GBS from 2009 to 2019 using the NSC data. Further, background mortality rates during the same period were estimated using data from Statistics Korea. Based on these data, the expected mortality rates for 2021 were estimated and compared with the observed mortality rates. |
| Limitations | 19 | Discuss limitations of the study, taking into account sources of potential bias or imprecision. Discuss both direction and magnitude of any potential bias | 25 | The limitations of this study were as follows. First, there were limitations to the claims data. Second, there are limitations to the data period. Third, there were some limitations to the methodology. |
| Interpretation | 20 | Give a cautious overall interpretation of results considering objectives, limitations, multiplicity of analyses, results from similar studies, and other relevant evidence | 25 | Although background rates are required to evaluate vaccine safety, comparing background rates with observed rates only suggests the possibility of an association between the vaccine and AEs and does not reveal a causal relationship. |
| Generalisability | 21 | Discuss the generalisability (external validity) of the study results | 25 | We used NSC and Statistics Korea data, which provide representation of the entire South Korean population to estimate the background rates of potential AEs following COVID-19 vaccination. |
| Other information | |  | | |
| Funding | 22 | Give the source of funding and the role of the funders for the present study and, if applicable, for the original study on which the present article is based | n/a |  |

*Give information separately for cases and controls in case-control studies and, if applicable, for exposed and unexposed groups in cohort and cross-sectional studies.

**Note:** An Explanation and Elaboration article discusses each checklist item and gives methodological background and published examples of transparent reporting. The STROBE checklist is best used in conjunction with this article (freely available on the Web sites of PLoS Medicine at http://www.plosmedicine.org/, Annals of Internal Medicine at http://www.annals.org/, and Epidemiology at http://www.epidem.com/). Information on the STROBE Initiative is available at www.strobe-statement.org.
