# Supplementary material for: COVID-19 vaccine safety: Background incidence rates of anaphylaxis, myocarditis, pericarditis, Guillain-Barré Syndrome, and mortality in South Korea using a nationwide population-based cohort study
Source: PLoS One. 2024 Feb 21;19(2):e0297902. doi: 10.1371/journal.pone.0297902 (PMC10881009; doi:10.1371/journal.pone.0297902)
Supplement: S1 Table — (DOCX) [file pone.0297902.s002.docx]

**Full Title**: COVID-19 vaccine safety: Background incidence rates of anaphylaxis, myocarditis, pericarditis, Guillain-Barré Syndrome, and mortality in South Korea using a nationwide population-based cohort study

**Short Title:** COVID-19 vaccine safety: Background rate

**Appendix file**

Table S1. Operational definition of events by ICD-10 code*

| **Events** | **ICD-code*** | |
| --- | --- | --- |
|  | inclusion criteria | exclusion criteria |
| **Anaphylaxis** | T78.2 (Anaphylactic-shock, unspecified), T80.5 (Anaphylactic shock due to serum), T88.6 (Anaphylactic shock due to adverse effect of correct drug or medicament properly administered | T63.4 (Venom of other arthropods), T78.0 (Anaphylactic shock due to adverse food reaction) |
| **Myocarditis** | I40.0 (Infective myocarditis), I40.1 (Isolated myocarditis), I40.8 (Other acute myocarditis), I40.9 (Acute myocarditis, unspecified), I41.0 (Myocarditis in bacterial diseases classified elsewhere), I41.1 (Myocarditis in viral diseases classified elsewhere), I41.2 (Myocarditis in other infectious and parasitic diseases classified elsewhere), I41.8 (Myocarditis in other diseases classified elsewhere), I51.4 (Myocarditis, unspecified) |  |
| **Pericarditis** | I30.0 (Acute nonspecific idiopathic pericarditis), I30.1 (Infective pericarditis), I30.8 (Other forms of acute pericarditis), I30.9 (Acute pericarditis, unspecified), I31.9 (Disease of pericardium, unspecified), I32.0 (Pericarditis in bacterial diseases classified elsewhere), I32.1 (Pericarditis in other infectious and parasitic diseases classified elsewhere) |  |
| **Guillain-Barré syndrome** | G61.0 (Guillain-Barré syndrome) |  |
| ICD-10: 10th revision of the International Classification Disease (ICD-10) | | |
