# Supplementary material for: COVID-19 vaccine safety: Background incidence rates of anaphylaxis, myocarditis, pericarditis, Guillain-Barré Syndrome, and mortality in South Korea using a nationwide population-based cohort study
Source: PLoS One. 2024 Feb 21;19(2):e0297902. doi: 10.1371/journal.pone.0297902 (PMC10881009; doi:10.1371/journal.pone.0297902)
Supplement: S2 Table — (DOCX) [file pone.0297902.s003.docx]

**Full Title**: COVID-19 vaccine safety: Background incidence rates of anaphylaxis, myocarditis, pericarditis, Guillain-Barré Syndrome, and mortality in South Korea using a nationwide population-based cohort study

**Short Title:** COVID-19 vaccine safety: Background rate

**Appendix file**

Table S2. Demographic characteristic of anaphylaxis cases

| Year | n (%) |
| --- | --- |
| **Total n (%)** | 1,041 (100.0%) |
| **Gender** |  |
| Men | 548 (52.6%) |
| Women | 493 (47.4%) |
| **Age group** |  |
| 0-19 | 108 (10.4%) |
| 20-29 | 72 ( 6.9%) |
| 30-39 | 108 (10.4%) |
| 40-49 | 174 (16.7%) |
| 50-59 | 258 (24.8%) |
| 60-69 | 185 (17.8%) |
| 70-79 | 109 (10.5%) |
| 80+ | 27 ( 2.6%) |
| **Health insurance type** |  |
| Health insurance | 990 (95.1%) |
| Medical aid | 51 ( 4.9%) |
| **Income quintile*** |  |
| First | 155 (14.9%) |
| Second | 138 (13.3%) |
| Third | 165 (15.9%) |
| Fourth | 249 (23.9%) |
| Fifth | 272 (26.1%) |
| missing or medical aid | 62 ( 6.0%) |
| *Income quintile: The first quintile represents the lowest 1/5 of values from 0-20% of the range. The second quintile includes the values from 20-40%, the third quintile includes 40-60%, the fourth quintile includes 60-80%, and the fifth quintile includes the highest 1/5 of values from 80-100%. | |
