# Supplementary material for: COVID-19 vaccine safety: Background incidence rates of anaphylaxis, myocarditis, pericarditis, Guillain-Barré Syndrome, and mortality in South Korea using a nationwide population-based cohort study
Source: PLoS One. 2024 Feb 21;19(2):e0297902. doi: 10.1371/journal.pone.0297902 (PMC10881009; doi:10.1371/journal.pone.0297902)
Supplement: S3 Table — (DOCX) [file pone.0297902.s004.docx]

**Full Title**: COVID-19 vaccine safety: Background incidence rates of anaphylaxis, myocarditis, pericarditis, Guillain-Barré Syndrome, and mortality in South Korea using a nationwide population-based cohort study

**Short Title:** COVID-19 vaccine safety: Background rate

**Appendix file**

Table S3. Crude incidence rate of anaphylaxis in 2009-2019

| Year | 2009 | | 2010 | | 2011 | | 2012 | | 2013 | | 2014 | |
| --- | --- | --- | --- | --- | --- | --- | --- | --- | --- | --- | --- | --- |
|  | CIR | 95% CI | CIR | 95% CI | CIR | 95% CI | CIR | 95% CI | CIR | 95% CI | CIR | 95% CI |
| **Total** | 4.15 | (2.94-5.47) | 4.07 | (2.85-5.40) | 3.99 | (2.77-5.33) | 3.92 | (2.68-5.26) | 6.12 | (4.56-7.78) | 6.26 | (4.69-7.92) |
| **Gender** |  |  |  |  |  |  |  |  |  |  |  |  |
| Men | 4.25 | (2.63-6.08) | 4.69 | (2.85-6.73) | 4.51 | (2.67-6.57) | 3.72 | (2.07-5.58) | 6.86 | (4.57-9.36) | 6.91 | (4.60-9.42) |
| Women | 4.05 | (2.43-5.87) | 3.46 | (1.83-5.29) | 3.48 | (1.84-5.32) | 4.11 | (2.47-5.96) | 5.38 | (3.51-7.44) | 5.61 | (3.53-7.90) |
| **Age group** |  |  |  |  |  |  |  |  |  |  |  |  |
| 0-19 | 0.49 | (0.00-1.47) | 1.57 | (0.00-3.67) | 2.27 | (0.57-4.54) | 1.24 | (0.00-3.10) | 4.10 | (1.37-7.52) | 3.04 | (0.76-6.08) |
| 20-29 | 3.47 | (0.69-6.94) | 0.00 | (0.00-0.00) | 0.73 | (0.00-2.19) | 0.74 | (0.00-2.21) | 1.48 | (0.00-3.70) | 2.94 | (0.73-5.87) |
| 30-39 | 2.92 | (0.58-5.84) | 2.96 | (0.59-5.91) | 1.20 | (0.00-3.00) | 3.64 | (1.21-6.68) | 2.48 | (0.62-4.96) | 3.18 | (0.64-6.35) |
| 40-49 | 5.59 | (2.24-9.51) | 3.93 | (1.12-7.30) | 7.30 | (3.37-11.79) | 3.93 | (1.12-7.31) | 6.12 | (2.78-10.02) | 3.89 | (1.11-7.23) |
| 50-59 | 5.98 | (2.24-10.47) | 4.90 | (1.40-9.11) | 5.28 | (1.98-9.24) | 5.74 | (2.55-9.57) | 9.30 | (4.96-14.25) | 11.49 | (6.65-16.93) |
| 60-69 | 11.90 | (4.76-20.23) | 5.83 | (1.17-11.66) | 8.12 | (2.32-15.09) | 6.81 | (2.27-12.48) | 16.48 | (8.79-25.27) | 11.52 | (5.24-18.85) |
| 70-79 | 3.83 | (0.00-9.57) | 12.80 | (3.66-23.78) | 5.19 | (0.00-12.12) | 9.68 | (3.23-17.75) | 4.71 | (0.00-10.98) | 13.83 | (6.15-23.06) |
| 80+ | 0.00 | (0.00-0.00) | 28.34 | (9.45-51.96) | 4.45 | (0.00-13.36) | 4.14 | (0.00-12.42) | 11.58 | (0.00-27.02) | 3.57 | (0.00-10.70) |
| CIR: Crude incidence rate; CI: confidence interval The crude incidence rate of anaphylaxis is expressed in episodes per 100,000 population. | | | | | | | | | | | | |
