# Supplementary material for: COVID-19 vaccine safety: Background incidence rates of anaphylaxis, myocarditis, pericarditis, Guillain-Barré Syndrome, and mortality in South Korea using a nationwide population-based cohort study
Source: PLoS One. 2024 Feb 21;19(2):e0297902. doi: 10.1371/journal.pone.0297902 (PMC10881009; doi:10.1371/journal.pone.0297902)
Supplement: S4 Table — (DOCX) [file pone.0297902.s005.docx]

**Full Title**: COVID-19 vaccine safety: Background incidence rates of anaphylaxis, myocarditis, pericarditis, Guillain-Barré Syndrome, and mortality in South Korea using a nationwide population-based cohort study

**Short Title:** COVID-19 vaccine safety: Background rate

**Appendix file**

Table S4. Demographic characteristic of myocarditis cases

| Year | n (%) |
| --- | --- |
| **Total n (%)** | 81 (100.0%) |
| **Gender** |  |
| Men | 53 (65.4%) |
| Women | 28 (34.6%) |
| **Age group** |  |
| 0-19 | 32 (39.5%) |
| 20-29 | 13 (16.0%) |
| 30-39 | 10 (12.3%) |
| 40-49 | 10 (12.3%) |
| 50-59 | 9 (11.1%) |
| 60-69 | 6 ( 7.4%) |
| 70+ | 1 ( 1.2%) |
| **Health insurance type** |  |
| Health insurance | 80 (98.8%) |
| Medical aid | 1 ( 1.2%) |
| **Income quintile*** |  |
| First | 14 (17.3%) |
| Second | 7 ( 8.6%) |
| Third | 17 (21.0%) |
| Fourth | 19 (23.5%) |
| Fifth | 23 (28.4%) |
| missing or medical aid | 1 ( 1.2%) |
| *Income quintile: The first quintile represents the lowest 1/5 of values from 0-20% of the range. The second quintile includes the values from 20-40%, the third quintile includes 40-60%, the fourth quintile includes 60-80%, and the fifth quintile includes the highest 1/5 of values from 80-100%. | |
