# Supplementary material for: COVID-19 vaccine safety: Background incidence rates of anaphylaxis, myocarditis, pericarditis, Guillain-Barré Syndrome, and mortality in South Korea using a nationwide population-based cohort study
Source: PLoS One. 2024 Feb 21;19(2):e0297902. doi: 10.1371/journal.pone.0297902 (PMC10881009; doi:10.1371/journal.pone.0297902)
Supplement: S5 Table — (DOCX) [file pone.0297902.s006.docx]

**Full Title**: COVID-19 vaccine safety: Background incidence rates of anaphylaxis, myocarditis, pericarditis, Guillain-Barré Syndrome, and mortality in South Korea using a nationwide population-based cohort study

**Short Title:** COVID-19 vaccine safety: Background rate

**Appendix file**

Table S5. Crude incidence rate of myocarditis in 2009-2019

| Year | 2009 | | 2010 | | 2011 | | 2012 | | 2013 | | 2014 | |
| --- | --- | --- | --- | --- | --- | --- | --- | --- | --- | --- | --- | --- |
|  | CIR | 95% CI | CIR | 95% CI | CIR | 95% CI | CIR | 95% CI | CIR | 95% CI | CIR | 95% CI |
| **Total** | 0.61 | (0.20-1.11) | 0.51 | (0.10-1.02) | 0.92 | (0.41-1.54) | 0.62 | (0.21-1.13) | 1.35 | (0.62-2.18) | 0.73 | (0.21-1.36) |
| Men | 0.61 | (0.00-1.42) | 0.61 | (0.00-1.43) | 1.44 | (0.41-2.67) | 0.83 | (0.21-1.65) | 2.08 | (0.83-3.54) | 1.26 | (0.42-2.30) |
| Women | 0.61 | (0.00-1.42) | 0.41 | (0.00-1.02) | 0.41 | (0.00-1.02) | 0.41 | (0.00-1.03) | 0.62 | (0.00-1.45) | 0.21 | (0.00-0.62) |
| **Age group** | | | | | | | | | | | | |
| 0-19 | 0.98 | (0.00-2.45) | 0.52 | (0.00-1.57) | 1.70 | (0.00-3.97) | 2.48 | (0.62-4.96) | 3.42 | (0.68-6.84) | 2.28 | (0.00-5.32) |
| 20-29 | 0.00 | (0.00-0.00) | 2.14 | (0.00-5.00) | 1.46 | (0.00-3.64) | 0.74 | (0.00-2.21) | 0.00 | (0.00-0.00) | 0.73 | (0.00-2.20) |
| 30-39 | 1.75 | (0.00-4.09) | 0.59 | (0.00-1.77) | 1.20 | (0.00-3.00) | 0.00 | (0.00-0.00) | 1.24 | (0.00-3.10) | 0.00 | (0.00-0.00) |
| 40-49 | 0.56 | (0.00-1.68) | 0.00 | (0.00-0.00) | 0.00 | (0.00-0.00) | 0.00 | (0.00-0.00) | 0.56 | (0.00-1.67) | 1.11 | (0.00-2.78) |
| 50-59 | 0.00 | (0.00-0.00) | 0.00 | (0.00-0.00) | 0.00 | (0.00-0.00) | 0.64 | (0.00-1.91) | 1.86 | (0.00-4.34) | 0.00 | (0.00-0.00) |
| 60-69 | 0.00 | (0.00-0.00) | 0.00 | (0.00-0.00) | 1.16 | (0.00-3.48) | 0.00 | (0.00-0.00) | 2.20 | (0.00-5.49) | 1.05 | (0.00-3.14) |
| 70+ | 0.00 | (0.00-0.00) | 0.00 | (0.00-0.00) | 1.73 | (0.00-5.19) | 0.00 | (0.00-0.00) | 0.00 | (0.00-0.00) | 0.00 | (0.00-0.00) |
| CIR: Crude incidence rate; CI: confidence interval The crude incidence rate of myocarditis is expressed in episodes per 100,000 population. | | | | | | | | | | | | |

**Table S5. Crude incidence rate of myocarditis in 2009-2019 (Continued)**

| Year | 2015 | | 2016 | | 2017 | | 2018 | | 2019 | |
| --- | --- | --- | --- | --- | --- | --- | --- | --- | --- | --- |
|  | CIR | 95% CI | CIR | 95% CI | CIR | 95% CI | CIR | 95% CI | CIR | 95% CI |
| **Total** | 0.52 | (0.10-1.05) | 0.95 | (0.42-1.58) | 0.85 | (0.32-1.49) | 0.75 | (0.21-1.39) | 0.65 | (0.22-1.18) |
| **Gender** |  |  |  |  |  |  |  |  |  |  |
| Men | 0.84 | (0.21-1.68) | 1.27 | (0.42-2.33) | 0.85 | (0.21-1.17) | 0.64 | (0.00-1.50) | 0.65 | (0.00-1.52) |
| Women | 0.21 | (0.00-0.63) | 0.63 | (0.00-1.47) | 0.85 | (0.21-1.69) | 0.85 | (0.21-1.70) | 0.64 | (0.00-1.50) |
| **Age group** |  |  |  |  |  |  |  |  |  |  |
| 0-19 | 0.85 | (0.00-2.56) | 4.86 | (0.97-9.71) | 4.49 | (1.12-8.99) | 1.32 | (0.00-3.95) | 4.75 | (0.00-11.08) |
| 20-29 | 1.46 | (0.00-3.64) | 0.72 | (0.00-2.16) | 0.72 | (0.00-2.15) | 1.43 | (0.00-3.58) | 0.00 | (0.00-0.00) |
| 30-39 | 0.00 | (0.00-0.00) | 0.00 | (0.00-0.00) | 0.00 | (0.00-0.00) | 0.68 | (0.00-2.04) | 0.70 | (0.00-2.10) |
| 40-49 | 0.56 | (0.00-1.68) | 1.13 | (0.00-2.83) | 0.57 | (0.00-1.71) | 0.59 | (0.00-1.76) | 0.59 | (0.00-1.78) |
| 50-59 | 0.60 | (0.00-1.79) | 0.59 | (0.00-1.77) | 1.17 | (0.00-2.94) | 0.58 | (0.00-1.73) | 0.00 | (0.00-0.00) |
| 60-69 | 0.00 | (0.00-0.00) | 0.00 | (0.00-0.00) | 0.00 | (0.00-0.00) | 0.83 | (0.00-2.50) | 0.79 | (0.00-2.36) |
| 70+ | 0.00 | (0.00-0.00) | 0.00 | (0.00-0.00) | 0.00 | (0.00-0.00) | 0.00 | (0.00-0.00) | 0.00 | (0.00-0.00) |
| CIR: Crude incidence rate; CI: confidence interval The crude incidence rate of myocarditis is expressed in episodes per 100,000 population. | | | | | | | | | | |
