# Supplementary material for: COVID-19 vaccine safety: Background incidence rates of anaphylaxis, myocarditis, pericarditis, Guillain-Barré Syndrome, and mortality in South Korea using a nationwide population-based cohort study
Source: PLoS One. 2024 Feb 21;19(2):e0297902. doi: 10.1371/journal.pone.0297902 (PMC10881009; doi:10.1371/journal.pone.0297902)
Supplement: S6 Table — (DOCX) [file pone.0297902.s007.docx]

**Full Title**: COVID-19 vaccine safety: Background incidence rates of anaphylaxis, myocarditis, pericarditis, Guillain-Barré Syndrome, and mortality in South Korea using a nationwide population-based cohort study

**Short Title:** COVID-19 vaccine safety: Background rate

**Appendix file**

Table S6. Demographic characteristic of pericarditis cases

| Year | n (%) |
| --- | --- |
| **Total n (%)** | 133 (100.0%) |
| **Gender** |  |
| Men | 78 (58.6%) |
| Women | 55 (41.4%) |
| **Age group** |  |
| 0-19 | 14 (10.5%) |
| 20-29 | 7 ( 5.3%) |
| 30-39 | 12 ( 9.0%) |
| 40-49 | 18 (13.5%) |
| 50-59 | 16 (12.0%) |
| 60-69 | 26 (19.5%) |
| 70-79 | 21 (15.8%) |
| 80+ | 19 (14.3%) |
| **Health insurance type** |  |
| Health insurance | 124 (93.2%) |
| Medical aid | 9 ( 6.8%) |
| **Income quintile*** |  |
| First | 23 (17.3%) |
| Second | 12 ( 9.0%) |
| Third | 18 (13.5%) |
| Fourth | 25 (18.8%) |
| Fifth | 44 (33.1%) |
| missing or medical aid | 11 ( 8.3%) |
| *Income quintile: The first quintile represents the lowest 1/5 of values from 0-20% of the range. The second quintile includes the values from 20-40%, the third quintile includes 40-60%, the fourth quintile includes 60-80%, and the fifth quintile includes the highest 1/5 of values from 80-100%. | |
