# Supplementary material for: COVID-19 vaccine safety: Background incidence rates of anaphylaxis, myocarditis, pericarditis, Guillain-Barré Syndrome, and mortality in South Korea using a nationwide population-based cohort study
Source: PLoS One. 2024 Feb 21;19(2):e0297902. doi: 10.1371/journal.pone.0297902 (PMC10881009; doi:10.1371/journal.pone.0297902)
Supplement: S7 Table — (DOCX) [file pone.0297902.s008.docx]

**Full Title**: COVID-19 vaccine safety: Background incidence rates of anaphylaxis, myocarditis, pericarditis, Guillain-Barré Syndrome, and mortality in South Korea using a nationwide population-based cohort study

**Short Title:** COVID-19 vaccine safety: Background rate

**Appendix file**

Table S7. Crude incidence rate of pericarditis in 2009-2019

| Year | 2009 | | 2010 | | 2011 | | 2012 | | 2013 | | 2014 | |
| --- | --- | --- | --- | --- | --- | --- | --- | --- | --- | --- | --- | --- |
|  | CIR | 95% CI | CIR | 95% CI | CIR | 95% CI | CIR | 95% CI | CIR | 95% CI | CIR | 95% CI |
| **Total** | 0.91 | (0.40-1.52) | 0.51 | (0.10-1.02) | 1.23 | (0.61-1.95) | 0.82 | (0.31-1.44) | 1.04 | (0.41-1.76) | 1.25 | (0.63-1.98) |
| **Gender** |  |  |  |  |  |  |  |  |  |  |  |  |
| Men | 1.01 | (0.20-2.03) | 0.82 | (0.20-1.63) | 1.85 | (0.82-3.08) | 0.83 | (0.21-1.65) | 1.04 | (0.21-2.08) | 1.67 | (0.63-2.93) |
| Women | 0.81 | (0.20-1.62) | 0.20 | (0.00-0.61) | 0.61 | (0.00-1.43) | 0.82 | (0.21-1.64) | 1.03 | (0.21-2.07) | 0.83 | (0.21-1.66) |
| **Age group** |  |  |  |  |  |  |  |  |  |  |  |  |
| 0-19 | 0.49 | (0.00-1.47) | 0.52 | (0.00-1.57) | 0.57 | (0.00-1.70) | 1.24 | (0.00-3.10) | 0.68 | (0.00-2.05) | 0.00 | (0.00-0.00) |
| 20-29 | 1.39 | (0.00-3.47) | 0.00 | (0.00-0.00) | 0.73 | (0.00-2.19) | 0.00 | (0.00-0.00) | 0.74 | (0.00-2.22) | 0.73 | (0.00-2.20) |
| 30-39 | 0.00 | (0.00-0.00) | 0.00 | (0.00-0.00) | 0.60 | (0.00-1.80) | 0.61 | (0.00-1.82) | 0.62 | (0.00-1.86) | 1.27 | (0.00-3.18) |
| 40-49 | 1.68 | (0.00-3.92) | 0.00 | (0.00-0.00) | 1.68 | (0.00-3.93) | 0.56 | (0.00-1.69) | 1.11 | (0.00-2.78) | 0.56 | (0.00-1.67) |
| 50-59 | 0.75 | (0.00-2.24) | 1.40 | (0.00-3.50) | 0.66 | (0.00-1.98) | 0.00 | (0.00-0.00) | 0.62 | (0.00-1.86) | 0.60 | (0.00-1.81) |
| 60-69 | 1.19 | (0.00-3.57) | 0.00 | (0.00-0.00) | 3.48 | (0.00-8.12) | 1.13 | (0.00-3.40) | 1.10 | (0.00-3.30) | 3.14 | (0.00-7.33) |
| 70-79 | 0.00 | (0.00-0.00) | 3.66 | (0.00-9.14) | 3.46 | (0.00-8.66) | 4.84 | (0.00-11.30) | 1.57 | (0.00-4.71) | 4.61 | (0.00-10.76) |
| 80+ | 5.07 | (0.00-15.21) | 0.00 | (0.00-0.00) | 0.00 | (0.00-0.00) | 0.00 | (0.00-0.00) | 7.72 | (0.00-19.30) | 3.57 | (0.00-10.70) |
| CIR: Crude incidence rate; CI: confidence interval The crude incidence rate of pericarditis is expressed in episodes per 100,000 population. | | | | | | | | | | | | |

**Table S7. Crude incidence rate of pericarditis in 2009-2019 (Continued)**

| Year | 2015 | | 2016 | | 2017 | | 2018 | | 2019 | |
| --- | --- | --- | --- | --- | --- | --- | --- | --- | --- | --- |
|  | CIR | 95% CI | CIR | 95% CI | CIR | 95% CI | CIR | 95% CI | CIR | 95% CI |
| **Total** | 1.05 | (0.42-1.78) | 1.48 | (0.74-2.32) | 1.91 | (1.06-2.87) | 1.92 | (1.07-2.89) | 1.83 | (0.97-2.80) |
| **Gender** |  |  |  |  |  |  |  |  |  |  |
| Men | 1.05 | (0.21-2.11) | 2.12 | (0.85-3.60) | 1.49 | (0.43-2.77) | 2.15 | (0.86-3.65) | 2.38 | (1.08-3.90) |
| Women | 1.04 | (0.21-2.09) | 0.84 | (0.21-1.68) | 2.32 | (1.06-3.80) | 1.70 | (0.64-2.98) | 1.28 | (0.43-2.35) |
| **Age group** |  |  |  |  |  |  |  |  |  |  |
| 0-19 | 0.85 | (0.00-2.56) | 1.94 | (0.00-4.86) | 1.12 | (0.00-3.37) | 2.63 | (0.00-6.58) | 3.17 | (0.00-7.91) |
| 20-29 | 0.00 | (0.00-0.00) | 0.72 | (0.00-2.16) | 0.00 | (0.00-0.00) | 0.00 | (0.00-0.00) | 0.72 | (0.00-2.15) |
| 30-39 | 1.29 | (0.00-3.23) | 0.66 | (0.00-1.98) | 1.35 | (0.00-3.37) | 1.36 | (0.00-3.41) | 0.00 | (0.00-0.00) |
| 40-49 | 1.12 | (0.00-2.81) | 1.13 | (0.00-2.83) | 0.57 | (0.00-1.71) | 1.17 | (0.00-2.93) | 0.59 | (0.00-1.78) |
| 50-59 | 0.00 | (0.00-0.00) | 1.18 | (0.00-2.96) | 1.17 | (0.00-2.94) | 1.16 | (0.00-2.89) | 2.30 | (0.57-4.60) |
| 60-69 | 0.97 | (0.00-2.92) | 2.76 | (0.00-6.45) | 4.38 | (0.88-8.75) | 3.34 | (0.83-6.67) | 3.14 | (0.79-6.29) |
| 70-79 | 1.52 | (0.00-4.57) | 1.50 | (0.00-4.49) | 4.32 | (0.00-10.07) | 2.78 | (0.00-6.96) | 4.07 | (0.00-9.49) |
| 80+ | 9.85 | (0.00-22.98) | 6.10 | (0.00-15.25) | 11.34 | (2.84-22.68) | 10.61 | (2.65-21.22) | 4.95 | (0.00-12.38) |
| CIR: Crude incidence rate; CI: confidence interval The crude incidence rate of pericarditis is expressed in episodes per 100,000 population. | | | | | | | | | | |
