# Supplementary material for: COVID-19 vaccine safety: Background incidence rates of anaphylaxis, myocarditis, pericarditis, Guillain-Barré Syndrome, and mortality in South Korea using a nationwide population-based cohort study
Source: PLoS One. 2024 Feb 21;19(2):e0297902. doi: 10.1371/journal.pone.0297902 (PMC10881009; doi:10.1371/journal.pone.0297902)
Supplement: S8 Table — (DOCX) [file pone.0297902.s009.docx]

**Full Title**: COVID-19 vaccine safety: Background incidence rates of anaphylaxis, myocarditis, pericarditis, Guillain-Barré Syndrome, and mortality in South Korea using a nationwide population-based cohort study

**Short Title:** COVID-19 vaccine safety: Background rate

**Appendix file**

Table S8. Demographic characteristic of Guillain–Barrè syndrome cases

| Year | n (%) |
| --- | --- |
| **Total n (%)** | 162 (100.0%) |
| **Gender** |  |
| Men | 88 (54.3%) |
| Women | 74 (45.7%) |
| **Age group** |  |
| 0-19 | 12 ( 7.4%) |
| 20-29 | 14 ( 8.6%) |
| 30-39 | 15 ( 9.3%) |
| 40-49 | 31 (19.1%) |
| 50-59 | 32 (19.8%) |
| 60-69 | 23 (14.2%) |
| 70-79 | 21 (13.0%) |
| 80+ | 14 ( 8.6%) |
| **Health insurance type** |  |
| Health insurance | 158 (97.5%) |
| Medical aid | 4 ( 2.5%) |
| **Income quintile*** |  |
| First | 22 (13.6%) |
| Second | 20 (12.3%) |
| Third | 33 (20.4%) |
| Fourth | 37 (22.8%) |
| Fifth | 44 (27.2%) |
| missing or medical aid | 6 ( 3.7%) |
| *Income quintile: The first quintile represents the lowest 1/5 of values from 0-20% of the range. The second quintile includes the values from 20-40%, the third quintile includes 40-60%, the fourth quintile includes 60-80%, and the fifth quintile includes the highest 1/5 of values from 80-100%. | |
