# Supplementary material for: COVID-19 vaccine safety: Background incidence rates of anaphylaxis, myocarditis, pericarditis, Guillain-Barré Syndrome, and mortality in South Korea using a nationwide population-based cohort study
Source: PLoS One. 2024 Feb 21;19(2):e0297902. doi: 10.1371/journal.pone.0297902 (PMC10881009; doi:10.1371/journal.pone.0297902)
Supplement: S9 Table — (DOCX) [file pone.0297902.s010.docx]

**Full Title**: COVID-19 vaccine safety: Background incidence rates of anaphylaxis, myocarditis, pericarditis, Guillain-Barré Syndrome, and mortality in South Korea using a nationwide population-based cohort study

**Short Title:** COVID-19 vaccine safety: Background rate

**Appendix file**

Table S9. Crude incidence rate of Guillain–Barrè syndrome (GBS) in 2009-2019

| Year | 2009 | | 2010 | | 2011 | | 2012 | | 2013 | | 2014 | |
| --- | --- | --- | --- | --- | --- | --- | --- | --- | --- | --- | --- | --- |
|  | CIR | 95% CI | CIR | 95% CI | CIR | 95% CI | CIR | 95% CI | CIR | 95% CI | CIR | 95% CI |
| Total | 0.81 | (0.30-1.42) | 1.12 | (0.51-1.83) | 0.92 | (0.41-1.54) | 1.34 | (0.62-2.16) | 1.35 | (0.62-2.18) | 1.77 | (0.94-2.71) |
| Gender |  |  |  |  |  |  |  |  |  |  |  |  |
| Men | 1.01 | (0.20-2.03) | 1.22 | (0.41-2.24) | 0.82 | (0.21-1.64) | 1.65 | (0.62-2.89) | 1.04 | (0.21-2.08) | 2.09 | (0.84-3.56) |
| Women | 0.61 | (0.00-1.42) | 1.02 | (0.20-2.03) | 1.02 | (0.20-2.05) | 1.03 | (0.21-2.06) | 1.65 | (0.62-2.89) | 1.45 | (0.42-2.70) |
| Age group |  |  |  |  |  |  |  |  |  |  |  |  |
| 0-19 | 0.49 | (0.00-1.47) | 1.05 | (0.00-2.62) | 0.00 | (0.00-0.00) | 1.24 | (0.00-3.10) | 0.68 | (0.00-2.05) | 0.76 | (0.00-2.28) |
| 20-29 | 1.39 | (0.00-3.47) | 0.71 | (0.00-2.14) | 0.00 | (0.00-0.00) | 0.00 | (0.00-0.00) | 1.48 | (0.00-3.70) | 0.73 | (0.00-2.20) |
| 30-39 | 0.58 | (0.00-1.75) | 0.59 | (0.00-1.77) | 0.00 | (0.00-0.00) | 1.21 | (0.00-3.04) | 0.62 | (0.00-1.86) | 3.18 | (0.64-6.35) |
| 40-49 | 1.68 | (0.00-3.92) | 1.69 | (0.00-3.93) | 0.56 | (0.00-1.68) | 1.69 | (0.00-3.93) | 0.56 | (0.00-1.67) | 0.00 | (0.00-0.00) |
| 50-59 | 0.75 | (0.00-2.24) | 2.10 | (0.00-4.90) | 0.66 | (0.00-1.98) | 3.19 | (0.64-6.38) | 2.48 | (0.62-4.96) | 1.81 | (0.00-4.23) |
| 60-69 | 0.00 | (0.00-0.00) | 0.00 | (0.00-0.00) | 5.80 | (1.16-11.61) | 1.13 | (0.00-3.40) | 1.10 | (0.00-3.30) | 3.14 | (0.00-7.33) |
| 70-79 | 0.00 | (0.00-0.00) | 1.83 | (0.00-5.49) | 3.46 | (0.00-8.66) | 0.00 | (0.00-0.00) | 4.71 | (0.00-10.98) | 1.54 | (0.00-4.61) |
| 80+ | 0.00 | (0.00-0.00) | 0.00 | (0.00-0.00) | 0.00 | (0.00-0.00) | 0.00 | (0.00-0.00) | 0.00 | (0.00-0.00) | 10.70 | (0.00-24.97) |
| CIR: Crude incidence rate; CI: confidence interval The crude incidence rate of GBS is expressed in episodes per 100,000 population. | | | | | | | | | | | | |

**Table S9. Crude incidence rate of Guillain–Barrè syndrome (GBS) in 2009-2019 (Continued)**

| Year | 2015 | | 2016 | | 2017 | | 2018 | | 2019 | |
| --- | --- | --- | --- | --- | --- | --- | --- | --- | --- | --- |
|  | CIR | 95% CI | CIR | 95% CI | CIR | 95% CI | CIR | 95% CI | CIR | 95% CI |
| **Total** | 1.47 | (0.73-2.31) | 2.22 | (1.37-3.17) | 2.02 | (1.17-2.97) | 2.46 | (1.50-3.53) | 1.51 | (0.75-2.37) |
| **Gender** |  |  |  |  |  |  |  |  |  |  |
| Men | 0.84 | (0.21-1.68) | 2.12 | (0.85-3.60) | 2.77 | (1.28-4.48) | 3.22 | (1.72-4.94) | 1.73 | (0.65-3.03) |
| Women | 2.09 | (0.84-3.55) | 2.31 | (1.05-3.78) | 1.27 | (0.42-2.32) | 1.70 | (0.64-2.98) | 1.28 | (0.43-2.35) |
| **Age group** |  |  |  |  |  |  |  |  |  |  |
| 0-19 | 0.85 | (0.00-2.56) | 1.94 | (0.00-4.86) | 1.12 | (0.00-3.37) | 1.32 | (0.00-3.95) | 0.00 | (0.00-0.00) |
| 20-29 | 0.73 | (0.00-2.18) | 1.44 | (0.00-3.61) | 1.43 | (0.00-3.58) | 1.43 | (0.00-3.58) | 0.72 | (0.00-2.15) |
| 30-39 | 0.00 | (0.00-0.00) | 0.66 | (0.00-1.98) | 1.35 | (0.00-3.37) | 1.36 | (0.00-3.41) | 0.00 | (0.00-0.00) |
| 40-49 | 1.12 | (0.00-2.81) | 1.13 | (0.00-2.83) | 3.42 | (1.14-6.28) | 2.93 | (0.59-5.86) | 2.97 | (0.59-5.94) |
| 50-59 | 1.79 | (0.00-4.18) | 2.96 | (0.59-5.91) | 2.35 | (0.59-4.70) | 1.16 | (0.00-2.89) | 0.57 | (0.00-1.72) |
| 60-69 | 0.97 | (0.00-2.92) | 5.53 | (1.84-10.13) | 0.88 | (0.00-2.63) | 2.50 | (0.00-5.84) | 1.57 | (0.00-3.93) |
| 70-79 | 7.62 | (1.52-15.25) | 2.99 | (0.00-7.48) | 1.44 | (0.00-4.32) | 5.57 | (1.39-11.14) | 2.71 | (0.00-6.78) |
| 80+ | 3.28 | (0.00-9.85) | 3.05 | (0.00-9.15) | 5.67 | (0.00-14.18) | 10.61 | (2.65-21.22) | 7.43 | (0.00-17.33) |
| CIR: Crude incidence rate; CI: confidence interval The crude incidence rate of GBS is expressed in episodes per 100,000 population. | | | | | | | | | | |
