# Supplementary material for: COVID-19 vaccine safety: Background incidence rates of anaphylaxis, myocarditis, pericarditis, Guillain-Barré Syndrome, and mortality in South Korea using a nationwide population-based cohort study
Source: PLoS One. 2024 Feb 21;19(2):e0297902. doi: 10.1371/journal.pone.0297902 (PMC10881009; doi:10.1371/journal.pone.0297902)
Supplement: S10 Table — (DOCX) [file pone.0297902.s011.docx]

**Full Title**: COVID-19 vaccine safety: Background incidence rates of anaphylaxis, myocarditis, pericarditis, Guillain-Barré Syndrome, and mortality in South Korea using a nationwide population-based cohort study

**Short Title:** COVID-19 vaccine safety: Background rate

**Appendix file**

Table S10. Demographic characteristic of death

| Year | n (%) |
| --- | --- |
| Total n (%) | 2,996,598 (100.0%) |
| Gender |  |
| Men | 1,643,195 (54.8%) |
| Women | 1,353,403 (45.2%) |
| Age group |  |
| 0-19 | 31,606 ( 1.1%) |
| 20-29 | 32,336 ( 1.1%) |
| 30-39 | 66,039 ( 2.2%) |
| 40-49 | 162,256 ( 5.4%) |
| 50-59 | 314,134 (10.5%) |
| 60-69 | 417,212 (13.9%) |
| 70-79 | 780,735 (26.1%) |
| 80+ | 1,192,280 (39.8%) |
