# Supplementary material for: COVID-19 vaccine safety: Background incidence rates of anaphylaxis, myocarditis, pericarditis, Guillain-Barré Syndrome, and mortality in South Korea using a nationwide population-based cohort study
Source: PLoS One. 2024 Feb 21;19(2):e0297902. doi: 10.1371/journal.pone.0297902 (PMC10881009; doi:10.1371/journal.pone.0297902)
Supplement: S11 Table — (DOCX) [file pone.0297902.s012.docx]

**Full Title**: COVID-19 vaccine safety: Background incidence rates of anaphylaxis, myocarditis, pericarditis, Guillain-Barré Syndrome, and mortality in South Korea using a nationwide population-based cohort study

**Short Title:** COVID-19 vaccine safety: Background rate

**Appendix file**

Table S11. Crude mortality rate in 2009-2019

| Year | 2009 | | 2010 | | 2011 | | 2012 | | 2013 | |
| --- | --- | --- | --- | --- | --- | --- | --- | --- | --- | --- |
|  | CR | 95% CI | CR | 95% CI | CR | 95% CI | CR | 95% CI | CR | 95% CI |
| Total | 497.20 | (495.24-499.16) | 511.90 | (509.92-513.89) | 513.61 | (511.63-515.60) | 530.68 | (528.67-532.69) | 526.56 | (524.56-528.56) |
| Gender |  |  |  |  |  |  |  |  |  |  |
| Men | 553.54 | (550.62-556.47) | 569.78 | (566.82-572.74) | 571.13 | (568.18-574.09) | 584.98 | (582.00-587.97) | 579.79 | (576.83-582.76) |
| Women | 440.64 | (438.03-443.26) | 453.85 | (451.21-456.50) | 455.97 | (453.33-458.62) | 476.31 | (473.61-479.01) | 473.30 | (470.62-475.98) |
| Age group |  |  |  |  |  |  |  |  |  |  |
| 0-19 | 31.43 | (30.43-32.45) | 31.09 | (30.09-32.11) | 30.29 | (29.28-31.30) | 29.24 | (28.24-30.25) | 27.21 | (26.23-28.19) |
| 20-29 | 56.84 | (55.10-58.59) | 54.28 | (52.54-56.02) | 51.42 | (49.72-53.14) | 44.98 | (43.37-46.60) | 42.31 | (40.74-43.89) |
| 30-39 | 92.13 | (90.09-94.18) | 87.37 | (85.36-89.38) | 83.16 | (81.19-85.14) | 79.54 | (77.61-81.48) | 78.23 | (76.30-80.17) |
| 40-49 | 206.54 | (203.52-209.56) | 201.69 | (198.72-204.68) | 187.69 | (184.82-190.57) | 180.51 | (177.70-183.34) | 174.38 | (171.62-177.16) |
| 50-59 | 432.96 | (427.84-438.10) | 420.77 | (415.88-425.68) | 408.17 | (403.50-412.85) | 389.87 | (385.42-394.33) | 382.13 | (377.79-386.49) |
| 60-69 | 1001.79 | (992.00-1011.60) | 971.68 | (962.15-981.23) | 922.96 | (913.74-932.22) | 873.55 | (864.63-882.49) | 821.85 | (813.33-830.39) |
| 70-79 | 2765.96 | (2744.97-2787.00) | 2740.95 | (2720.60-2761.33) | 2623.60 | (2604.21-2643.03) | 2610.77 | (2592.00-2629.57) | 2475.49 | (2457.69-2493.33) |
| 80+ | 9398.67 | (9333.29-9464.17) | 9423.86 | (9360.71-9487.13) | 9245.11 | (9184.62-9305.70) | 9466.63 | (9407.43-9525.92) | 8902.56 | (8847.26-8957.95) |
| CR: Crude mortality rate; CI: confidence interval The crude mortality rate is expressed per 100,000 population. | | | | | | | | | | |

| Year | 2014 | | 2015 | | 2016 | | 2017 | | 2018 | |
| --- | --- | --- | --- | --- | --- | --- | --- | --- | --- | --- |
|  | CR | 95% CI | CR | 95% CI | CR | 95% CI | CR | 95% CI | CR | 95% CI |
| Total | 527.25 | (525.25-529.25) | 541.40 | (539.38-543.42) | 549.34 | (547.31-551.37) | 557.27 | (555.23-559.31) | 582.40 | (580.31-584.49) |
| Gender |  |  |  |  |  |  |  |  |  |  |
| Men | 580.50 | (577.54-583.47) | 590.89 | (587.90-593.87) | 597.47 | (594.47-600.47) | 603.33 | (600.32-606.34) | 629.49 | (626.42-632.56) |
| Women | 474.03 | (471.36-476.71) | 491.99 | (489.27-494.72) | 501.33 | (498.59-504.07) | 511.35 | (508.58-514.12) | 535.49 | (532.66-538.32) |
| Age group |  |  |  |  |  |  |  |  |  |  |
| 0-19 | 27.16 | (26.18-28.16) | 24.81 | (23.86-25.77) | 25.06 | (24.10-26.03) | 23.22 | (22.28-24.17) | 22.66 | (21.71-23.62) |
| 20-29 | 39.18 | (37.68-40.70) | 39.67 | (38.16-41.19) | 37.41 | (35.95-38.89) | 36.60 | (35.16-38.05) | 37.26 | (35.82-38.72) |
| 30-39 | 75.70 | (73.77-77.63) | 70.57 | (68.69-72.45) | 68.79 | (66.92-70.68) | 66.47 | (64.61-68.33) | 69.78 | (67.86-71.71) |
| 40-49 | 165.83 | (163.15-168.52) | 161.23 | (158.57-163.89) | 153.55 | (150.96-156.16) | 147.01 | (144.46-149.56) | 148.20 | (145.62-150.79) |
| 50-59 | 372.41 | (368.19-376.65) | 352.85 | (348.78-356.94) | 346.94 | (342.92-350.96) | 332.43 | (328.52-336.36) | 330.72 | (326.84-334.62) |
| 60-69 | 781.67 | (773.54-789.81) | 758.53 | (750.77-766.30) | 740.85 | (733.43-748.28) | 692.58 | (685.59-699.58) | 684.90 | (678.12-691.68) |
| 70-79 | 2358.00 | (2340.87-2375.17) | 2333.62 | (2316.73-2350.55) | 2239.04 | (2222.59-2255.52) | 2138.00 | (2122.17-2153.86) | 2092.56 | (2077.20-2107.94) |
| 80+ | 8597.45 | (8545.16-8649.82) | 8584.69 | (8534.62-8634.84) | 8393.18 | (8345.56-8440.87) | 8342.71 | (8297.01-8388.47) | 8407.70 | (8363.42-8452.04) |
| CR: Crude mortality rate; CI: confidence interval The crude mortality rate is expressed per 100,000 population. | | | | | | | | | | |

**Table S11. Crude mortality rate in 2009-2019 (Continued)**

**Table S11. Crude mortality rate in 2009-2019 (Continued)**

| Year | 2019 | |
| --- | --- | --- |
|  | CR | 95% CI |
| Total | 574.71 | (572.63-576.78) |
| Gender |  |  |
| Men | 625.85 | (622.79-628.91) |
| Women | 523.80 | (521.00-526.60) |
| Age group |  |  |
| 0-19 | 21.87 | (20.92-22.83) |
| 20-29 | 37.75 | (36.29-39.22) |
| 30-39 | 68.97 | (67.05-70.91) |
| 40-49 | 143.00 | (140.43-145.56) |
| 50-59 | 320.36 | (316.57-324.17) |
| 60-69 | 652.44 | (646.01-658.89) |
| 70-79 | 1948.12 | (1933.52-1962.76) |
| 80+ | 7833.72 | (7792.52-7874.98) |
| CR: Crude mortality rate; CI: confidence interval The crude mortality rate is expressed per 100,000 population. | | |
